# Leucine-Rich Repeats and Transmembrane Domain 2 Controls Protein Sorting in the Striatal Projection System and Its Deficiency Causes Disturbances in Motor Responses and Monoamine Dynamics

**DOI:** 10.3389/fnmol.2022.856315

**Published:** 2022-05-09

**Authors:** Misato Ichise, Kazuto Sakoori, Kei-ichi Katayama, Naoko Morimura, Kazuyuki Yamada, Hiroki Ozawa, Hayato Matsunaga, Minoru Hatayama, Jun Aruga

**Affiliations:** ^1^Department of Medical Pharmacology, Nagasaki University Graduate School of Biomedical Sciences, Nagasaki, Japan; ^2^Department of Neuropsychiatry, Nagasaki University Graduate School of Biomedical Sciences, Nagasaki, Japan; ^3^Laboratory for Behavioral and Developmental Disorders, RIKEN Brain Science Institute (BSI), Wako-shi, Japan; ^4^Support Unit for Animal Experiments, RIKEN Brain Science Institute (BSI), Wako-shi, Japan

**Keywords:** Lrtm2, gene targeting, striatal projection neuron, behavioral abnormality, monoamine metabolism, protein sorting

## Abstract

The striatum is involved in action selection, and its disturbance can cause movement disorders. Here, we show that leucine-rich repeats and transmembrane domain 2 (Lrtm2) controls protein sorting in striatal projection systems, and its deficiency causes disturbances in monoamine dynamics and behavior. The Lrtm2 protein was broadly detected in the brain, but it was enhanced in the olfactory bulb and dorsal striatum. Immunostaining revealed a strong signal in striatal projection output, including GABAergic presynaptic boutons of the SNr. In subcellular fractionation, Lrtm2 was abundantly recovered in the synaptic plasma membrane fraction, synaptic vesicle fraction, and microsome fraction. Lrtm2 KO mice exhibited altered motor responses in both voluntary explorations and forced exercise. Dopamine metabolite content was decreased in the dorsal striatum and hypothalamus, and serotonin turnover increased in the dorsal striatum. The prefrontal cortex showed age-dependent changes in dopamine metabolites. The distribution of glutamate decarboxylase 67 (GAD67) protein and gamma-aminobutyric acid receptor type B receptor 1 (GABA_*B*_R1) protein was altered in the dorsal striatum. In cultured neurons, wild-type Lrtm2 protein enhanced axon trafficking of GAD67-GFP and GABA_*B*_R1-GFP whereas such activity was defective in sorting signal-abolished Lrtm2 mutant proteins. The topical expression of hemagglutinin-epitope-tag (HA)-Lrtm2 and a protein sorting signal abolished HA-Lrtm2 mutant differentially affected GABA_*B*_R1 protein distribution in the dorsal striatum. These results suggest that Lrtm2 is an essential component of striatal projection neurons, contributing to a better understanding of striatal pathophysiology.

## Introduction

The striatum constitutes the largest nucleus of the basal ganglia and comprises the dorsal striatum (caudate and putamen) and ventral striatum (nucleus accumbens). The dorsal striatum is included in the neural circuit that regulates the initiation of voluntary movements, and the ventral striatum is involved in the cognitive processing of motivation, aversion, reward, and reinforcement learning (Hall and White, 2018).

Neural circuits in the striatum are known as below (Gerfen and Surmeier, 2011; Gerfen and Bolam, 2016; Supplementary Figure 1). All cortical areas, including the motor cortex and thalamus, form glutamatergic inputs to the striatum, forming synaptic connections with GABAergic spiny projection neurons (SPN) and four classes of interneurons. SPNs convey striatal information flow to the rest of the basal ganglia through direct and indirect pathways. Direct pathway SPNs extend their axons to the internal segment of the internal segment of globus pallidus (GPi) and substantia nigra pars reticulata (SNr). Indirect pathway SPNs extend their axons to the external segment of the external segment of globus pallidus (GPe). Direct pathway neurons also send their axon collaterals to the GPe. GPe neurons (GABAergic) project to the subthalamic nucleus (STN) and output nuclei (GPi/SNr). STN neurons (glutamatergic) project to the GPi and GPi/SNr, forming a pathway parallel to the direct pathway. GPi and SNr neurons project to the thalamus, superior colliculus, and pedunculopontine nuclei.

SPNs also project to dopaminergic neurons in the SNc, inhibiting the release of dopamine (DA) that is conveyed along the nigrostriatal pathway to SPNs in the dorsal striatum. DA acts on DA receptors in SPNs and decreases the inhibitory outflow of the basal ganglia (Hall and White, 2018).

In basal ganglia neural circuits, SPN projections converge greatly on GPi/SNr. In humans, it is estimated that more than 100 SPNs innervate globus pallidus (GP) cells, where individual SPN axons are in sparse contact with many pallidal neurons before terminating densely in the axons of target neurons (Hall and White, 2018). The functional significance of the striatonigral projections is suggested by the striatonigral degeneration that occurs as a subtype of multiple systemic atrophy (MSA), a progressive neurodegenerative disorder (Krismer and Wenning, 2017). In MSA with predominant parkinsonism (MSA-P), neuronal loss is most pronounced in the striatonigral system (Krismer and Wenning, 2017). Parkinsonism, with rigidity, slow movement, postural instability, gait disability, and tendency to fall, characterizes the poorly levodopa-responsive motor presentation of MSA-P (Krismer and Wenning, 2017). Although the morphological features and clinical importance of the striatal projection system have been well recognized, the molecular basis underlying its proper function has not been fully understood. In particular, the machinery that controls nigrostriatal protein trafficking is still limited.

SPNs of the direct pathway are known to express the GABA-synthesizing enzyme GAD67 (Gad1) and GABA_*B*_ receptor1 (GABA_*B*_R1, Gabbr1) genes, and their gene products are detected in the axon terminals of SNr (Biermann et al., 2010; Tritsch et al., 2014). Mechanisms underlying axon transport of these molecules have been investigated (Kanaani et al., 1999; Biermann et al., 2010; Carrel et al., 2011), and altered axon transport has been implicated in neurological disorders (Rocco et al., 2016; Dinamarca et al., 2019). However, no mechanisms selective to striatonigral projections are known.

In mouse SPN, Leucine-rich repeats and transmembrane domains 2 (Lrtm2) protein is strongly distributed as will be shown later. The functions and biological roles of Lrtm2 have not been reported. However, many transmembrane proteins with Leucine-rich repeat (LRR) domains have been shown to play critical roles in neurite and synapse control in the nervous system (Aruga and Mikoshiba, 2003; Lauren et al., 2003; Dolan et al., 2007; Ko, 2012; Schroeder and de Wit, 2018). Some of them are involved in the pathogenesis of neurological disorders (Abelson et al., 2005; Tekin et al., 2013). These circumstances led us to investigate the biological role of Lrtm2. We firstly investigated Lrtm2 protein distribution and behavioral abnormalities appeared in Lrtm2 knockout (KO) mice in an unbiased manner. Its intense distribution in SPN and SNr as well as abnormalities motor responses led us to further examine the monoamine content and molecular marker disturbances in Lrtm2 KO mice. Based on these results, we hypothesized a role of Lrtm2 in protein sorting, tested the hypothesis using ectopic expression of mutant Lrtm2 proteins *in vitro* and *in vivo*, and discussed the pathophysiological significance of the findings.

## Materials and Methods

### Bioinformatics Analysis

Homology search and ortholog search was done at NCBI databases^1^^2^ or ENSEMBL database.^3^ Protein motif search was done at Expasy.^4^ Signal sequence prediction was done at SignalP.^5^ Sequence alignment was edited by MEGA7 (Kumar et al., 2016).

### Animals

All animal experiments were approved by the Animal Experiment Committee of RIKEN and Animal Care and Use Committee of Nagasaki University (Approval number 1803271441) and were carried out following the guidelines for Animal Experimentation at RIKEN and Nagasaki University. CD-1 mice and Sprague-Dawley rats were purchased from Japan SLC and CLEA Japan.

### Generation of Leucine-Rich Repeats and Transmembrane Domains 2 Null Mutant Mice

Lrtm2 null mutant mice were generated as previously described (Katayama et al., 2010). Briefly, to construct the Lrtm2 targeting vector, overlapping Lrtm2 genomic clones were isolated from a BAC library made of 129 SV strain mice. The targeting construct contained the 3.1-kb 5′ and 9.1-kb 3′ homology regions, and the 9.2-kb fragment containing the open reading flame of Lrtm2 was replaced with the phosphoglycerol kinase (PGK)-neo expression cassette flanked by a loxP sequence. E14 embryonic stem (ES) cells were electroporated and selected using G418. Drug-resistant clones were analyzed by Southern blotting. *Eco*RI-or *Spe*I-digested genomic DNA was hybridized with a 0.7-kb 5’ genomic fragment that corresponded to the genomic sequence outside the target vector or a 0.6-kb *Pst*I PGK-neo probe, respectively. Chimeric mice were generated by injecting targeted ES cells into C57BL/6J blastocysts. To eliminate the PGK-neo cassette, germline-transmitted mice were first mated with mice transgenic for a Cre recombinase gene under the control of the cytomegalovirus immediate early enhancer-chicken β-actin hybrid (CAG) promoter (Sakai and Miyazaki, 1997). Mice carrying the mutated Lrtm2 allele were backcrossed to C57BL/6J for more than six generations before analysis. Genotyping of the progeny was performed by Southern blotting or PCR analysis of DNA from the tail. The PCR primers used were Lrtm2S (5′-GTTCTGGCTCAACACCTCATAG-3′), Lrtm2WTAS (5′-GATTTATAACTCCCGCAGGTCAG-3′), and Lrtm2KOAS (5′-GCTTTATCACCCTTGTCCTGAG-3′).

### Antibodies

**FIGURE 1:**
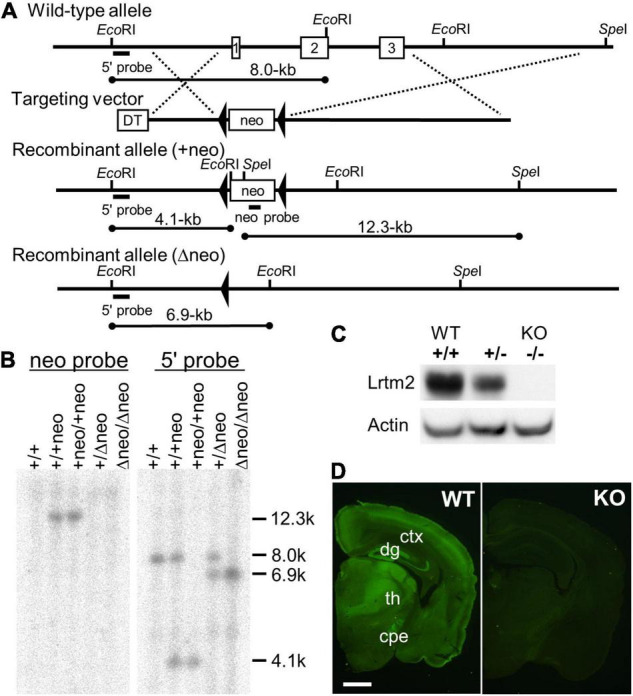
Targeted disruption of the Lrtm2 gene. **(A)** Structures of the Lrtm2 genomic locus, targeting vector, and mutated allele. The locations of the probes for Southern blotting (5’ and neo probes) are shown. DT, diphteria toxin A; neo, neomycin-resistance gene. **(B)** Confirmation of homologous recombination of the mutant alleles by Southern blot. **(C)** Western blot performed on proteins prepared from brains of adult Lrtm2+/+ (WT), Lrtm2+/-, and Lrtm2−/− (KO) mice. **(D)** Immunostaining of Lrtm2 KO brain using anti-Lrtm2 antibody. 8 weeks-old. WT, littermate control. cpe, cerebral peduncle; ctx, cerebral cortex; dg, dentate gyrus of hippocampus; th, thalamus. *Scale bar*, 1 mm.

Polyclonal anti-Lrtm2 antibody was raised in a rabbit against peptides corresponding to the carboxy-terminal region of mouse Lrtm2 (KRQPLMGDPEGEHEDQKQISSVA). Peptides were synthesized and conjugated to keyhole limpet hemocyanin through cysteine added to the N-terminus of the peptide. After immunization with conventional methods, antisera were obtained, and the antibody was purified by affinity chromatography with the immunized peptide. The validity of the antibody was confirmed by the absence of the corresponding signals in Lrtm2 KO-derived samples by immunoblotting and immunostaining (Figures 1C,D). Information for the other antibodies is shown in Supplementary Table 1.

### RNA Analysis

Northern blotting and *in situ* hybridization were performed as previously described (Aruga and Mikoshiba, 2003). For quantitative PCR (qPCR) analysis, brain punch regions and circular tissue punches were taken from the dorsal striatum, GP, and SNr using disposable biopsy needles (Biopsy Punch; Kai Medical) with diameters of 1.0, 1.0, and 0.5 mm, respectively, from 150 μm-thick frozen coronal brain sections. RNA was isolated using TRIzol reagent (Thermo Fisher). cDNA was synthesized using SuperScript II reverse transcriptase (Thermo Fisher). Calibration curves were generated commonly by using a whole brain cDNA. Real-time RT-PCR analysis was carried out using Power SYBR Green PCR Master Mix (Thermo Fisher) and ABI PRISM 7900HT (Thermo Fisher). Primer sequences are available upon request.

### Immunoblotting

Specimens were homogenized in RIPA buffer [50 mM Tris-HCl pH 8.0, 150 mM sodium chloride, 1% NP-40, 0.5% sodium deoxycholate, 0.1% SDS, 1 mM EDTA, and complete protease inhibitor cocktail (Roche Diagnostics, Mannheim, Germany)]. For subcellular fractionation, whole brain homogenates were fractionated by differential centrifugation, followed by postsynaptic density fractionation as previously described (Tomioka et al., 2014). The extracted proteins were loaded onto SDS-PAGE gels, electrophoresed, and transferred to a polyvinylidene fluoride membrane (Millipore, Billerica, MA). Signals were visualized using an ECL or ECL Plus kit (GE Healthcare, Buckinghamshire, United Kingdom). Full blot images are shown in Supplementary Figure 3. For subcellular fractionation analysis, the blotted membrane was cut into two pieces along 75 kDa marker position. The piece containing higher molecular weight proteins was reacted with mouse anti-PSD95. The piece with lower molecular weight proteins was reacted with mouse anti-Synaptophysin antibody, followed by reaction with rabbit anti-Lrtm2 antibody after deactivating horse radish peroxidase by immersing the membrane in 0.1% sodium azide in phosphate buffered saline (PBS) for 10 min.

### Immunostaining

Mice were anesthetized with inhalation of isoflurane or intraperitoneal injection of 0.75 mg/kg of medetomidine, 4.0 mg/kg of midazolam, and 5.0 mg/kg of butorphanol. Cardiac perfusion was performed with 4% paraformaldehyde and 0.1 M sodium phosphate (pH 7.4) at a rate of 6.5 mL/min for 3 min. Excised brains were fixed in the same fixative for 4 h at 20–25°C with gentle agitation. Tissue blocks (3-mm thick) were prepared using a razor blade and Rodent Brain Matrix (ASI Instruments) and cryoprotected with 20% sucrose in PBS at 4°C overnight. After adding the OCT compound (Sakura Finetek) to 65% (v/v), the tissue blocks were placed in the resultant embedding medium for 15 min with gentle agitation. The blocks were placed in Cryomold (Sakura Finetek) with the embedding medium and rapidly frozen on dry ice or prechilled aluminum blocks in a −80°C freezer. Cryosection was performed using a CM3050 cryostat (Leica Biosystems) at a thickness of either 12 or 10 μm. After sectioning, the sections were blocked with 2% normal goat serum and 0.1% Triton X-100 in PBS and reacted with primary antibodies at 4°C for 0.5–4 days, followed by appropriate fluorescence-labeled secondary antibodies at room temperature for 1 h. The stained sections were mounted under glass coverslips with Vectashield with 4’,6-diamidino-2-phenylindole (DAPI) (Vector Laboratories). For double labeling with anti-GABA_*B*_R1 antibody, the sections were treated with HistoVT One (Nacalai Tesque, Kyoto, Japan) for 20 min at 70°C before antibody reaction. Fluorescence images of whole sections were obtained using a fluorescence microscope (BZ-X800, Keyence). For quantitative immunostaining of brain sections, confocal images were obtained using a microscope (FV1000, Olympus; LSM800 Zeiss) with a 60 × objective. For quantitative immunostaining of cultured neurons, the fluorescence images were scanned using an LSM800 (Zeiss) with a 20 × or 63 × objective. For quantitative analyses, all stained images were taken with the same laser settings, and the fluorescence intensity was quantified using ImageJ software with the same parameters. In the particle analysis for a mouse, 2 or 6 ROIs (212 μm × 212 μm) from independent sections were subjected to quantify the particles with 0.3–1.5 μm^2^ areas, and mean values were used for the statistical analysis.

### Behavioral Analysis

Adult Lrtm2 KO and WT mice (2–6-month-old male littermates from mated heterozygotes) were used for behavioral tests unless otherwise noted. Male mice were used to avoid effects of estrous cycles on behavioral phenotypes in females (Meziane et al., 2007). The mice were 2–3 months of age at the start of behavioral testing, and were tested starting with less stressful behavioral tasks. Mice were housed in a 12:12 h light-dark cycle, with the dark cycle occurring from 20:00 to 8:00, and behavioral experiments were carried out between 10:00 and 17:00. Home cage activity measurement, open field test, light-dark box test, elevated plus-maze test, fear conditioning test, forced swimming test, acoustic startle response, marble burying test, novel object test, social interaction in the open field test, and resident intruder test were performed as described in previous studies (Katayama et al., 2010; Takashima et al., 2011).

#### Hole Board Test

A box made of gray plastic [50 × 50 × 40 (H) cm] with four equally separated holes (3-cm in diameter with an infrared sensor) on the floor was used (Model ST-1/WII, Muromachi-kikai, Tokyo, Japan). The field was illuminated by fluorescent light (180 lx at the center of the field), and the level of background noise was approximately 50 dB. The behavior of each mouse was monitored by a CCD camera located approximately 1.5 m above the field. In the hole board test, the mice were individually introduced into the center of the field and allowed to explore freely for 5 min. The total moving time (s), distance traveled (cm), latency of head-dipping (s), number of head-dips, duration of head-dipping (s), duration of rearing (s), and number of rearings were measured as indices. Data were collected and analyzed using the CompACT VAS system (Muromachi-Kikai, Tokyo, Japan).

#### Social Discrimination Test

This test was performed in an open field test apparatus with a luminance of 70 lx. The test consisted of the first session (a habituation session with empty cages), the second session (a test session with one caged mouse), and the third session (a test session with two caged mice). Each session lasted 15 min and occurred in the following order. In the habituation session, two empty cylindrical wire cages [inner size, 7 cm φ × 15 cm (H); outer size, 9 cm φ × 16.5 cm (H), with 21 vertical stainless (3-mm-φ wires) longitudinally and gray polyvinyl discs on the top and bottom, manufactured by the RIKEN Rapid Engineering Team] were placed in two adjacent corners. In the second session, a mouse (7-week-old male DBA2, purchased from Nihon SLC, Shizuoka, Japan) that was new to the test mouse was placed in one of two cylindrical cages. In the third session, another mouse that was also new to the test mouse was placed in the remaining cylindrical cage. Between the three sessions, there were 4-min intervals during which the test mouse was returned to its home cage. The three sessions were video-recorded from above, and the times spent in the two corner squares containing the cylinders within the 3 × 3-square subdivision (17.7 × 17.7 cm^2^) were measured with Image J OF4 (O’Hara). For the two test sessions, video recording was also performed from an obliquely upward position to observe contact between the test mouse and the in-cage mouse. Contact with the in-cage mouse was defined as a forward movement toward the mouse in the cage and subsequent direct contact with the head. The position and posture of the in-cage mice were observed through the slits of the wires. The contacts were counted on the video records by an observer who was blind to the genotypes. Each in-cage mouse was used once a day, and when the habituation session began, the mouse was simultaneously placed in its cylindrical cage on the corners of an open field box that was not being used for the tests. These rules were thought to minimize the difference between the two in-cage mice in the second test session concerning their acclimatization to the cylindrical cage and the open field box environment. After each use, the cylindrical cage was extensively washed with water and rinsed with 90% ethanol, which was then evaporated to minimize the effects of the remaining materials.

#### Rotarod Test

Rotarod evaluations were performed for both young (3–5-month-old) and aged (15 months) mice using a Rotarod Treadmill MK-610A Muromachi apparatus. Briefly, one habituation session was conducted 24 h before the beginning of the task, consisting of one practical trial of 2 min (4 rpm). The mice were then subjected to the paradigm for 4 consecutive days, each one consisting of four rotarod trials (with 1-min intervals between trials, allowing the animals to rest but avoiding them to become inactive). The rod accelerated from 4 to 40 rpm over 240 s and was maintained at 40 rpm for 60 s thereafter. The maximum trial duration was 300 s. Animals that fell off the rod or failed to turn one full revolution were returned to the cage. The set of 4 consequtive days trials was carried out twice with 4 days interval.

#### Tail Suspension Test

The tail suspension test was conducted as previously described (Wesolowska and Nikiforuk, 2007). Mice were attached to a wire using an adhesive tape placed approximately 1.5 cm from the tip of the tail and suspended 30 cm above the floor. The duration of immobility was recorded for 5 min.

#### Morris Water Maze Test

A circular maze made of white plastic (1-m diameter, 30-cm depth) was filled with water to a depth of approximately 20 cm (22–23°C). The water was colored by adding white paint to prevent the mice from seeing the platform (20 cm high, 10 cm diameter; 1 cm below the surface of the water) or other cues under the water. Some extra maze landmark cues (i.e., a calendar, figure, and plastic box) were visible to the mice in the maze. The movements of the mice in the maze were recorded and analyzed using Image J WM (O’Hara). Mice received six trials (one session) per day for 4 consecutive days. Each acquisition trial was initiated by placing each mouse into the water facing the outer edge of the maze at one of four designated starting points quasi-randomly, and the position of the submerged platform remained constant for each mouse throughout the testing. The trial was terminated when the mouse reached the platform, and the latency and distance swum were measured. The cut-off time for the trial was 60 s; mice that did not reach the platform within 60 s were removed from the water and placed on the platform for 30 s before being toweled off and placed back into their home cages. The intertrial interval was approximately 6 min. After 4 days of training, a probe test was conducted on day 5. In the probe test, the platform was removed, and each mouse was placed in the water at a point opposite the target platform and allowed to swim in the maze for 60 s. The distance swum, the number of crossings of the position of the target platform and the other three platforms, and the time spent in each of the four quadrants were measured.

### Neurochemical Analysis

Brains from 5-month-old mice (young group) and 13–20-month-old mice (aged group) were collected immediately after decapitation, and 150 μm-thick frozen coronal sections were prepared. Circular tissue punches were collected from the medial prefrontal cortex, hippocampus, basolateral, and cortical nuclei of the amygdala, dorsal striatum, and hypothalamus using disposable biopsy needles (Biopsy Punch; Kai Medical). The samples were homogenized in 0.1 M perchloric acid containing 0.1 mM EDTA and centrifuged for 15 min at 20,000 × g at 4°C. The supernatant was then filtered through 0.22 μm PVDF micropore filters (Millipore), and the filtrate was analyzed by high-performance liquid chromatography (HPLC) coupled to an electrochemical detection system (graphite electrode vs. Ag/AgCl reference, Eicom). Briefly, a Prepak AC-ODS 4.0 × 5.0 mm precolumn and an Eicompak SC-5ODS 3.0 × 150 mm column were used for separation, which was a mobile phase consisting of 44.7 mM citrate, 40.3 mM sodium acetate, 15% methanol, 190 mg/L sodium 1-octanesulfonate, and 5 mg/L EDTA, adjusted to pH 3.7 using glacial acetic acid and pumped at a rate of 0.5 mL/min. The working electrode (WE-3G) potential was set at 0.5 V. The column temperature was maintained at 25°C. The HPLC data were collected automatically and analyzed using EZChrom Elite (Scientific Software). Protein levels were measured using a DC protein assay kit (Bio-Rad). All analyte information, including the retention times, peak heights, concentration, and recovery rate of the internal standards, was calculated concerning standard curves generated for known concentrations of external standards run daily.

### Plasmid Construction and Recombinant Adeno-Associated Virus Vector Preparation

Mouse Lrtm2, Gad1, and Gabbr1 cDNAs were PCR-cloned from mouse brain cDNA (Takara Bio), optionally epitope-tagged, and sequenced. Lrtm2 protein sorting sequence mutants have been generated by *Dpn*I-mediated site-directed mutagenesis (Fisher and Pei, 1997). For transfection into hippocampal neurons, the above DNA fragments were inserted into pCAGGS (Niwa et al., 1991), pcDNA3.1 (Thermo Fisher Scientific), or pEGFP-N3 (Takara Bio). The GAD67: GFP expression plasmid was constructed by inserting the Gad1 cDNA without a termination codon inserted between the *Sal*I-*Apa*I sites of pEGFP-N3 and the remaining G-P-G-S-I-A-T as a linker peptide sequence between Gad1 and GFP. The GABA_*B*_R1a:GFP expression plasmid was constructed by inserting Gabbr1 cDNA without a termination codon inserted into the *Bam*HI site of pEGFP-N3 (Takara Bio), and the remaining G-S-I-A-T as a linker peptide sequence between GABA_*B*_R1 and GFP. For viral expression constructs, the tdTomato coding sequence of pAAV-CAG-tdTomato (codon diversified, a gift from Edward Boyden Addgene plasmid #59462; RRID:Addgene_59462)^6^ was replaced with the HA-Lrtm2WT, HA-Lrtm2mAD, or GAD67:GFP sequence. Recombinant adeno-associated virus (AAV) preparation, purification, and titration were performed as previously described (Challis et al., 2019).

### Cell Culture and Transfection

Hippocampal neurons were isolated from embryonic day 18 rat brains and cultured in a neurobasal medium supplemented with B27 (Invitrogen) at the density of 1.25 × 10^4^ cells/well of 24-well dish as described in a previous study (Morimura et al., 2017). Transfection was performed 7 days after using Lipofectamine2000 (Invitrogen). At least three independent culture experiments were done to collect the well separated neurons with sufficient neurites. For immunofluorescence staining, cells were fixed with PFA fixative 4 days after the transfection, blocked with 2% normal goat serum (Jackson ImmunoResearch), 0.1% TritonX100, PBS, and reacted with primary antibodies. Axons were identified by anti-neurofilament monoclonal antibody cocktails (SMI312, Biolegend) and dendrites were identified by anti-MAP2 antibody (AP-20, Sigma). To measure the neurites area, MAP2 or SMI312-immunopositive signals within 15 μm distant from the center of target neuron cell body was removed. Then, the areas of the remaining MAP2 or SMI312-immunopositive regions were measured by ImageJ function. Neurite length and dendrite complexity was measured by using Neurolucida software (MBF Bioscience).

### Adeno-Associated Virus Injection and Analysis

Recombinant AAV injection was performed as described (Stoica et al., 2013) using a stereotaxic apparatus (SR-6M-HT, Narishige) and a microliter syringe (Hamilton). Mice were anesthetized with an intraperitoneal injection of 0.75 mg/kg of medetomidine, 4.0 mg/kg of midazolam, and 5.0 mg/kg of butorphanol. The dorsal striatum was targeted to the following coordinates by bregma: AP, −1.0 mm; ML, + 2.0 mm; DV, + 3.5 mm. Two microliters of virus solution (AAV-HA-Lrtm2WT and AAV-HA-Lrtm2mAD, 1.8 × 10^12^ virus genome/mL; AAV-GAD67: GFP, 7.5 × 10^11^ virus genome/mL) was injected at a rate of 0.5 μL/min. The needle tip was kept in the injected position for 1 min and kept in AP, −1.0 mm; ML, + 2.0 mm; DV, + 2.5 mm for 4 min. The injected mice were fixed by cardiac perfusion and immersion, as described above, 12 days after injection. Immunostaining analysis was performed as described previously. The age and sex of the mice were matched by using littermates between the groups to compare.

### Experimental Design and Statistical Analyses

Data are expressed as means with standard deviations (SD) otherwise stated. Differences were considered statistically significant at *p* < 0.05. *p*-values are those obtained by statistical tests between WT and KO mice (*n* = mouse number) otherwise stated. The sample sizes for each experiment were determined such that the power and significance in the two-sided test were 80 and 5%, respectively (Festing, 2018). However, the number of samples from the animals was minimized empirically. The Student’s two-tailed unpaired *t*-test, two-tailed paired *t* -test, Welch’s two-tailed unpaired *t*-test, or Mann-Whitney *U*-test was used to determine the statistical significance of differences between the two groups. Student’s two-tailed unpaired *t*-test was used unless otherwise stated. Kolmogorov-Smirnov test for normal distribution was carried out to judge whether parametric or non-parametric tests should be used. To compare the effects of mutations on Lrtm2 properties, one-way ANOVA and *post hoc* Dunnett test was performed. To examine the influence of the two independent categorical variables, a two-way repeated-measures ANOVA was performed. Statistical analyses were performed using Microsoft Excel (Microsoft), SPSS statistical package (ver. 16, SPSS Inc.), and BellCurve for Excel (Social Survey Research Information Co., Ltd.).

## Results

### Leucine-Rich Repeats and Transmembrane Domains 2 Gene and Its Products

**FIGURE 2:**
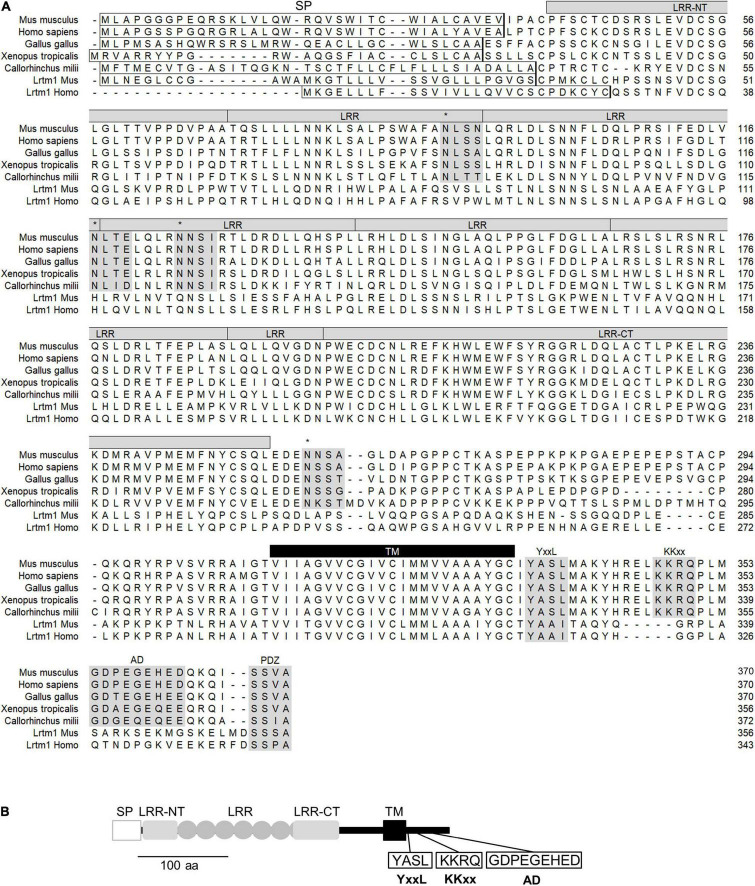
Structure of Lrtm2 protein. **(A)** Sequence alignment and conserved domains of Lrtm2 proteins. *N-terminal box*, predicted signal peptide sequence; *shaded*, conserved sequence motifs. *asterisks*, N-glycosylation sites; LRR, Leucine-rich repeat; LRR-NT, LRR-N-terminal; LRR-CT, LRR-C-terminal; YxxL, endocytosis motif; KKxx, ER retrieving motif; AD, acidic domain known as a motif for axon targeting and KIF1-binding; PDZ, PDZ domain-binding; TM, transmembrane domain. **(B)** Domain structure of Lrtm2 protein. SP, signal peptide; LRR-NT and LRR-CT, cap structures of LRR domains; ellipse, a unit of Leucine-rich repeat; TM, transmembrane domain. YxxL, somatodendritic sorting signal; KKxx, ER-retrieval signal; AD, acidic residue clustered domain.

Lrtm2 is a gene conserved in vertebrates (Figure 2A). Lrtm2 encodes a type I transmembrane protein of 370 aa in humans and mice (Figure 2A), which is relatively small compared to the other LRR-transmembrane protein families (Dolan et al., 2007). The LRR domain is located between the N-terminal signal sequence and the transmembrane domain and includes six repeated LRR motifs and capping structures at both terminals (LRR-NT and LRR-CT) (Figure 2). Lrtm2 does not belong to Lrrtm family proteins, which are known as synaptic adhesion molecules (Ko, 2012; Schroeder and de Wit, 2018), in terms of the domain organization and the protein sizes (Dolan et al., 2007). In the presumptive cytoplasmic domain, there were some candidate sequences for protein sorting (Figure 2). These included YxxL/Yxxφ (endocytosis-associated/somatodendritic targeting) (Li et al., 2000), KKxx (ER retrieving signal) (Townsley and Pelham, 1994), and acidic domain (AD) (axon targeting) (Kratchmarov et al., 2013).

**FIGURE 3:**
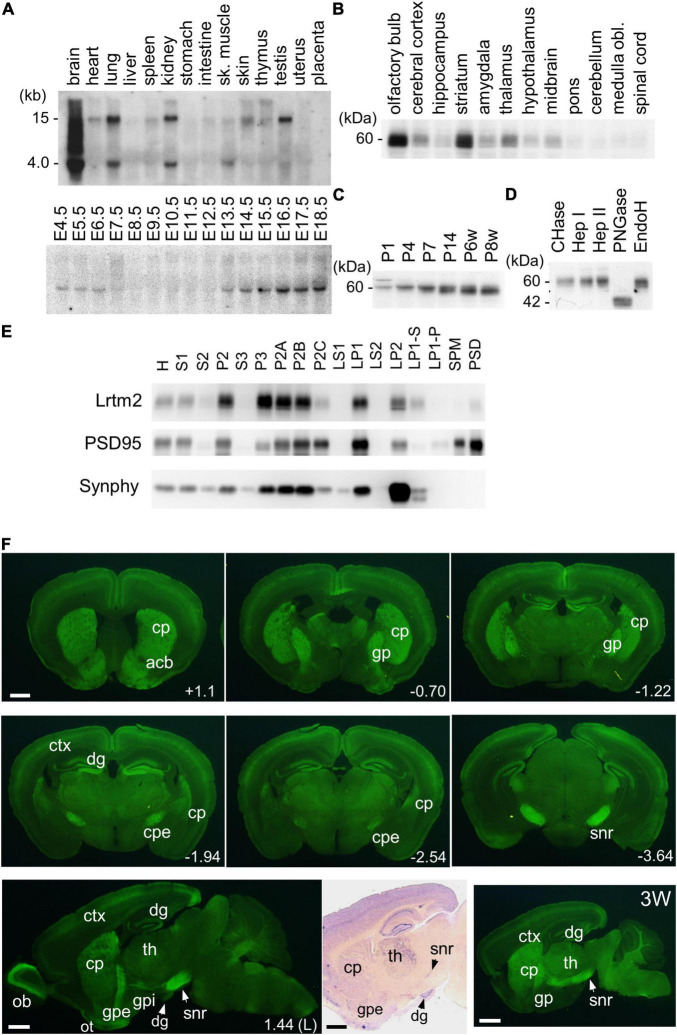
Expression of Lrtm2 and distribution of Lrtm2 protein. **(A)** Northern blot analysis. *Top*, organ blot. *Bottom*, temporal expression profile during embryonic development. sk, skeletal; E, embryonic day. **(B–E)** Immunoblot analysis. **(B)** Regional distribution of Lrtm2 in the brain. **(C)** Lrtm2 abundance in the brain during postnatal development. P, postnatal day or week (w). **(D)** Glycosidase sensitivity of Lrtm2 protein. Only peptide N-glycosidase F (PNGase) resulted in a band shift to a lower molecular weight range, indicating that Lrtm2 is an N-glycosylated protein. CHase, chondroitinase ABC; Hep I, heparinase I; Hep II, heparinase II; EndoH, endoglycosidase H. **(E)** Subcellular distribution. H, homogenates; S1, postnuclear supernatant; S2, supernatant after P2 precipitation; P2, crude synaptosome; S3, cytosol; P3, light membrane; P2A, myelin-enriched fraction; P2B, synaptosome-enriched fraction; P2C, mitochoindria-enriched fraction; LS1, supernatant after LP1 precipitation; LP1, crude synaptosomal membranes; LS2, supernatant after LP2 precipitation; LP2, synaptic vesicle-enriched fraction; LP1-S, 1% Triton-X100-soluble LP1 fraction; LP1-P, 1% Triton-X100-insoluble LP1 fraction; SPM, synaptosomal plasma membranes; PSD, postsynaptic density fraction; PSD95, an excitatory postsynaptic protein; Synphy, synaptophysin, a presynaptic marker. **(F)** Immunostaining. 8-weeks-old or 3-weeks-old (3W) mouse brain sections are immunostained with an anti-Lrtm2 antibody. cp, caudoputamen; cpe, cerebral peduncle; ctx, cerebral cortex; dg, dentate gyrus of hippocampus; gp, globus pallidus; gpe, external segment of globus pallidus; gpi, internal segment of globus pallidus; ob, olfactory bulb; ot, olfactory tubercle; snr, substantia nigra pars reticulata; th, thalamus. *Numerical values* in the pictures indicate relative distance (mm) from Bregma or midsagittal plane (L) (Paxinos and Franklin, 2001). *Bright field image*, *in situ* hybridization showing Lrtm2 mRNA distribution in 1.44 (L) parasagittal section. Note the difference when compared with Lrtm2 protein (left adjacent image). *Scale bar*, 1 mm.

First, we examined the distribution of Lrtm2 mRNA in mice. In an adult organ, it was strongly detected in the brain and weakly in the lung, kidney, and testes, while a faint distribution was observed in organs other than the placenta (Figure 3A). In contrast, the Lrtm2 related gene Lrtm1 was faintly detected in the brain (Supplementary Figure 2). In developing embryos, Lrtm2 mRNA increased during E13.5-E18.5 (Figure 3A).

Next, we raised an antibody against mouse Lrtm2 and examined the properties of the Lrtm2 protein in immunoblots (Figures 3B–E). In the adult mouse brain, the Lrtm2 protein was strongly detected in the olfactory bulb, striatum, cerebral cortex, hippocampus, amygdala, thalamus, hypothalamus, and midbrain regions, and weakly in the others (Figure 3B). The abundance of the whole brain increased during postnatal development (Figure 3C). Lrtm2 is a glycosylated protein but is not a proteoglycan, as indicated by its sensitivity to peptide N-glycosidase F treatment and by its resistance to chondroitinase ABC, heparinase I, heparinase II, and endoglycosidase H treatments (Figure 3D). In a subcellular fractionation experiment (Figure 3E), the Lrtm2 protein was recovered in membrane fractions (P2 and P3), predominantly in synaptosomal plasma membrane fractions (LP1, LP2) and in the microsomal fraction (P3). Recovery from the LP1 (synaptosomal membrane fraction) and LP2 (synaptic vesicle enriched fraction) suggested a localization at both plasma membrane and internal compartments like recycling endosomes or synaptic vesicles.

### Distribution of the Leucine-Rich Repeats and Transmembrane Domains 2 Protein and mRNA

**FIGURE 4:**
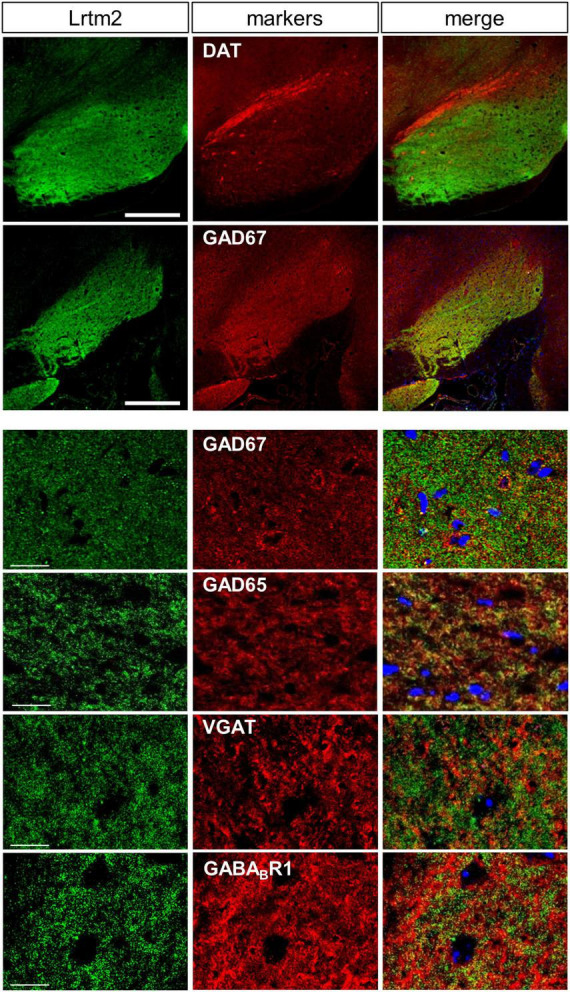
Distribution of Lrtm2 in SNr. All pictures derive from double immunostaining of SNr in parasagittal sections with Lrtm2 (*green*) and indicated markers (*red*). Lrtm2 and GAD67 are colocalized except for local inhibitory neurons in which the cell body is marked with strong GAD67 signals surrounding the nuclei (*blue*, DAPI), as seen in panels with higher magnification. *Scale bars*, 500-μm-thick; 20-μm-thin.

We then examined the distribution of the Lrtm2 protein by immunostaining. In the adult mouse brain, the strongest immunopositive signals were observed in the SNr (Figures 3F, 4). The other striatal projection systems (striatum, GPe, and GPi) also produced strong signals. Moderate to strong signals were observed in the thalamic nuclei. In the cerebral cortex, there were moderate signals throughout, but the signals were enhanced in the deep layer (layer 4) (Supplementary Figure 4). The cerebral peduncle showed strong signals (Figure 3F). In the hippocampus, dentate gyrus cells, and mossy fibers of the granule neurons gave the signals (Figures 1, 3F and Supplementary Figure 4). In the olfactory bulb, both the outer and inner plexiform layers produced strong signals (Figure 3F). The olfactory tubercle also provided signals (Figure 3F).

There were differences in the distribution between the Lrtm2 immunopositive signals (Figure 3F) and mRNA signals that can be seen at bright field image in Figure 3F and images deposited in Allen Brain Atlas database (Lein et al., 2007). Lrtm2 mRNA signals were the strongest in the thalamus. In the striatal projection systems, mRNA signals were observed in the striatum, but not in the SNr, GPe, GPi, or STN (Figure 3F and Table 1). In the cerebral cortex, Lrtm2 mRNA was distributed in layers 2 and 3, whereas the immunopositive signals were strong in layer 4 (Figure 3F, Table 1, and Supplementary Figure 4). In the olfactory bulb, Lrtm2 mRNA was strongly in the granule cell layer and scarce in the outer and inner plexiform layers in an opposite manner to the Lrtm2 protein (Figure 3F and Table 1). When we compared the Lrtm2 mRNA and protein distribution (Table 1), it became clear that Lrtm2 proteins are abundant at the sites where Lrtm2 mRNA-positive neurons extend their axons. This relationship is commonly observed in striatal projection systems, cortical projections (cerebral cortex to cerebral peduncle), thalamocortical projections (thalamus to cerebral cortex layer 4), and olfactory bulb (granule neuron to plexiform layers). We hypothesized that Lrtm2 mRNA is mainly translated in soma or dendrites, and some fraction of the Lrtm2 protein was preferentially transported to the axon and axon terminals.

**TABLE 1:** Comparison of Lrtm2 mRNA and Lrtm2 protein distribution in mouse brain.

Region	Structure	Lrtm2 mRNA	Lrtm2 protein
Olfactory bulb			
	Glomerular layer	+	
	Outer plexiform layer		++
	Mitral cell layer	+	
	Inner plexiform layer		+
	Granule cell layer	++	
Cerebral cortex		All layer	Enhanced at IV, weakly at II/III
	Frontal pole	+	
	Anterior cingulate	++	
	Infralimbic	++	
	Motor	++	+
	Somatosensory	+	+
	Visual	+	+
	Retrosplenial	+	+ 1)
	Piriform cortex II	+	
	Olfactory tubercle II	++	
	Entorhinal cortex	+	
Midbrain	Cerebral peduncle		++
Hippocampus			
	Dentate gyrus		
	Molecular layer		+
	Granule cell layer	++	
	Hilus		+
	CA3		+
Thalamus			
	Ventral posterolateral/posteromedial nuclei	++	+
	Reticular nucleus, medical/lateral habenula	-	+ 2)
	Lateral geniculate complex	++	
	Medial geniculate complex	++	
	Posterior complex	+	+
	Paraventricular nucleus	++ 3)	-
	Parataenial nucleus	++	
	Anterodorsal/anteroventral nuclei	++	+
	Anteromedial nucleus	+	
	Intermediodorsal nucleus	+	-
	Mediodorsal nucleus	+	
	Central medial nucleus	+	
	Rhomboid nucleus	+	
	Submedial nucleus	+	
	Paracentral nucleus	+	
	Central lateral nucleus	+	
	Lateral posterior nucleus	-	+
	Nucleus of reuniens	+	
	Lateral dorsal nucleus	++	+ 3)
	Peripeduncular nucleus	++	
Basal ganglia			
	Dorsal striatum (caudate and putamen)	++	++
	Ventral striatum (nucleus accumbens)	+	+
	Globus pallidus, external segment		++
	Globus pallidus, internal segment		+
	Substantia nigra reticular part		++
Cerebellum			
	Purkinje cell layer	+	
	Deep cerebellar nuclei (interpositus)		+
Medulla			
	Torapezoid body	+	
	Medial vestibular nucleus	+	+
	Lateral vestibular nucleus		+
	External cuneate nucleus	+	+
	Dorsal motor nucleus of vagus nerve	+	
	Medullary reticular nucleus, ventral part	+	

*Protein distribution is based on this study, and mRNA distribution is based on Allen brain atlas (https://mouse.brain-map.org/, 69838338, 74819475). (1) Immunopositive signals are also observed at layer I; (2) lateral habenula; (3) dorsal part.*

Lrtm2 protein distribution was further examined at higher magnification in SNr, where a dense distribution of immunopositive signals was observed. Among synaptic marker proteins, Lrtm2 immunopositive signals overlapped well with those of GAD65 (Gad2) and GAD67 (Gad1), partly with VGAT but not with gephyrin, VGLUT1, or VGLUT2 (Figure 4). In SNr, Lrtm2 signals were distributed in a reticular pattern where VGAT-positive punctate signals were surrounded by strong Lrtm2 signals (Figure 4). It is known that SNr includes dendrites of dopaminergic neurons that extend from the SNc. Lrtm2 signals were not overlapped with, but were located adjacent to dopaminergic dendrites in the ventral tiers of SNc (Figure 4). These results suggested that Lrtm2 protein was localized in axon terminals of SPNs throughout SNr without enhancement on the synaptic vesicles.

### Behavioral Abnormalities of Leucine-Rich Repeats and Transmembrane Domains 2 Knockout Mice

To determine the role of Lrtm2 *in vivo*, we generated the Lrtm2 null type mutation allele (*Lrtm2*^–^) by replacing the entire Lrtm2 coding sequence with a loxP sequence (Figure 1). Abnormalities of male homozygotes (*Lrtm2*^–^*^/^*^–^, KO) were explored by comparing them with wild-type littermates (*Lrtm2^+/+^*, WT) in behavioral test batteries (Table 2).

**TABLE 2:** Summary of Lrtm2 KO behavioral analysis.

Test		Result	Statistical significance and notes	WT	KO
Home-cage activity	No. of count by infrared sensor	No change		7	10
Open field (dark)	Locomotor	No change		10	10
	% Time in center	No change		10	10
Open field (bright)	Locomotor	No change		10	10
	% time in center	No change		10	10
Light-dark box	% time in light box	No change		7	10
	No. of transitions	Slightly decreased	*p* = 0.073	7	10
	Latency entering dark box	No change		7	10
	Total distance	No change		7	10
Elevated plus maze	% Time in open arm	No change		7	10
	% Entries to open arm	No change		7	10
	Total distance	No change		7	10
Hole-board	Head-dip latency	Increased	*p = 0.011 (Welch’s *t*-test)	10	10
	Total distance	No change		10	10
	Rearing number	No change		10	10
	Head-dip time	Decreased	***p = 0.00035	10	10
	Head-dip number	Decreased	*p = 0.014	10	10
Staircase	Latency	Slightly increased	*p* = 0.062	7	10
	No. of step ascend	No change		7	10
Water maze	Latency reaching goal	No change		10	10
	Probe test of time in quadrant	No change		10	10
	Probe test of crossing	No change		10	10
	Moving speed	Decreased	**,*day1–3, *p* = 0.0024, 0.0079, 0.032	10	10
	No movement time	Increased	**day1–2, *p* = 0.0099, 0.0095	10	10
Fear conditioning—conditioning	Freezing response before unconditioned stimulus	No change		7	10
	freezing response after unconditioned stimulus (tone + footshock)	No change		7	10
Fear conditioning—context	freezing response	No change		7	10
Fear conditioning—cue	Freezing response before conditioned stimulus	No change		7	10
	Freezing response after conditioned stimulus (tone cue)	No change		7	10
Rotarod (3-5 M-old)	Time stayed on rotating rod	No change		16	18
Rotarod (15 M-old)	Time stayed on rotating rod	Increased at later stage (day 9–12, mean)	*p = 0.021	16	16
Tail suspension	Immobility time	Decreased	*p = 0.013 (*U*-test)	10	13
Forced swimming	Immobility time	No change		11	16
Hotplate	Latency licking forepaws	No change		10	10
	Latency flinching	No change		10	10
Tail flick	Latency removing tail	No change		10	10
Acoustic startle response	Startle response amplitude	No change		7	10
	Prepulse inhibition	No change		7	10
Marble burying	No. of marbles buried	No change		16	18
Novel object	Approach in homecage	Slightly decreased	*p* = 0.075	16	18
	Approach time in open field	Slightly decreased	*p* = 0.054	10	10
	Approach count in open field	Decreased	**p = 0.0036	10	10
Social interaction	Particles in novel environment	No change		10	10
Social discrimination	Session1	No change		16	18
	Session2	Reduced approach to mice in cage	*p = 0.029	16	18
	Session3	Reduced approach to mice in cage	*p = 0.045 (near zone-stayed time, decreased)	16	18
			*p = 0.028 (far zone-stayed time, increased)		
Resident intruder	Nose to anogenital contact	No change		16	18
	Nose to head contacting	No change		16	18
Body weight		Decreased	(−6.6%) **p = 0.0020	17	20

Statistical significance indicates the difference between WT and KO, *p < 0.05; **p < 0.01; ***p < 0.001 in Student’s unpaired two-tailed t-test unless otherwise indicated.

**FIGURE 5:**
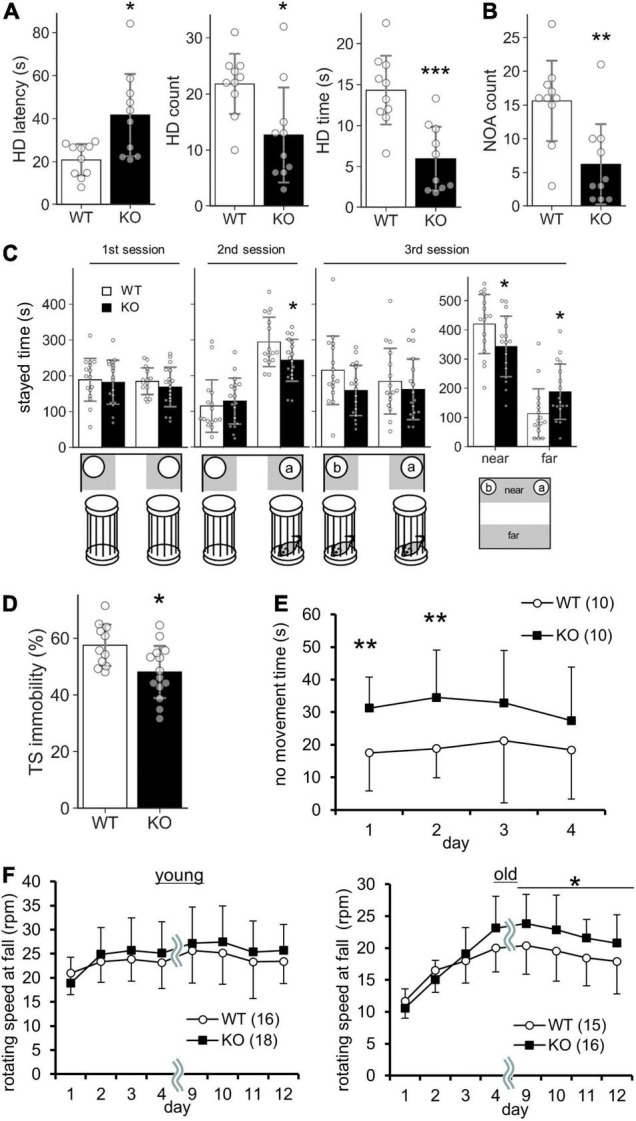
Behavioral abnormalities of Lrtm2 KO mice. 2–6-month-old male were subjected for systematic behavioral analysis. Results for additional parameters for the above tests and additional test subjects are indicated in Table 2. **(A)** Hole board test. Mice are placed in an open field box with holes for 5 min. Left, head dip (HD) time latency. Middle, HD count. Right, total HD time. WT, *n* = 10, KO, *n* = 10. **(B)** Novel object approach (NOA) test. Mice are placed in an open field with an unfamiliar object (inverted paper cup). The number of contacts is counted. WT, *n* = 10, KO, *n* = 10. **(C)** Social discrimination test. Bottom, experimental design. Graphs indicate the time spent in the gray areas indicated below. *near*, the region close to the caged mice; *far*, the region distant from the caged mice. WT, *n* = 10, KO, *n* = 10. **(D)** Tail suspension test. The percentage of immobile time in 5 min test period is measured. WT, *n* = 10, KO, *n* = 13. **(E)** No movement time in the water maze test. WT, *n* = 10, KO, *n* = 10. **(F)** Rotarod test. The tests are carried out daily on days 1–4 and days 9–12. The stayed time on accelerating rotarod is measured. WT, *n* = 16, KO, *n* = 16–18. *p < 0.05; **p < 0.01; ***p < 0.001 in Welch’s *t* -test (1A, HD latency), *U*-test **(D)**, and Student’s *t*-test (the others). *Error bars*, SD. **(A–D)** *Gray circles* indicate the individual value of each mouse. **(E,F)** The numbers in parentheses indicate *n* (the number of mice) in each experimental group.

Lrtm2 KO mice did not show abnormalities in locomotor activity in their home cage or the open field test (Table 2). However, when they were placed in an open field box with four holes (hole board test), KO mice took a longer time before investigating any holes (*t* = 3.05, *p* = 0.011, Welch’s *t*-test) and spent a shorter time investigating the holes (*t* = 4.39, *p* = 0.00035) (Figure 5A). The count of the approach to an inanimate novel object in an open field box was also reduced (*t* = 3.50, *p* = 0.0036) (Figure 5B). In a social discrimination test, KO mice spent a shorter time near the cylinder cage with a mouse (2nd-session, *t* = 2.28, *p* = 0.029, *t*-test; 3rd-session-near, *t* = 2.09, *p* = 0.045) (Figure 5C). Taken together, these results suggest that the Lrtm2 KO mice exhibited altered exploratory behavior.

Lrtm2 KO mice also showed abnormalities in behavioral tasks that forced movement. In the tail suspension test, the immobility was reduced (*U* = 25, *p* = 0.013, *U*-test) (Figure 5D). In the Morris water maze test, no movement time was increased [*F*_(1_, _79)_ = 5.33, p_*genotype*_ = 0.033, two-way repeated-measures ANOVA for genotype and trials as main factors], but the movement time and moving speed in the swimming pool were not increased without any abnormalities in spatial learning-related parameters (Figure 5E and Table 2). In the rotarod test performed for both 3–5-month-old and 15-month-old mice, 15-month-old Lrtm2 KO mice stayed longer on the rotating rod (day 9–12, mean, *t* = 29, *p* = 0.021) (Figure 5F and Table 2).

Other behavioral tasks, including the elevated plus maze, light-dark box test, elevated plus maze test, fear conditioning test, forced swimming test, hot plate test, tail-flick test, acoustic startle response test, marble burying test, and social interaction tests for freely moving animal objects did not show any obvious abnormalities, but the body weight of Lrtm2 KO mice was slightly lower than that of WT mice (−6.6%, *t* = 3.35, *p* = 0.0020) (Table 2). Overall, the behavioral abnormalities in Lrtm2 KO mice were believed to reflect altered voluntary movement initiation, motor learning, and exploratory behaviors.

### Brain Monoamine Contents Were Altered in Leucine-Rich Repeats and Transmembrane Domains 2 Knockout Mice

**FIGURE 6:**
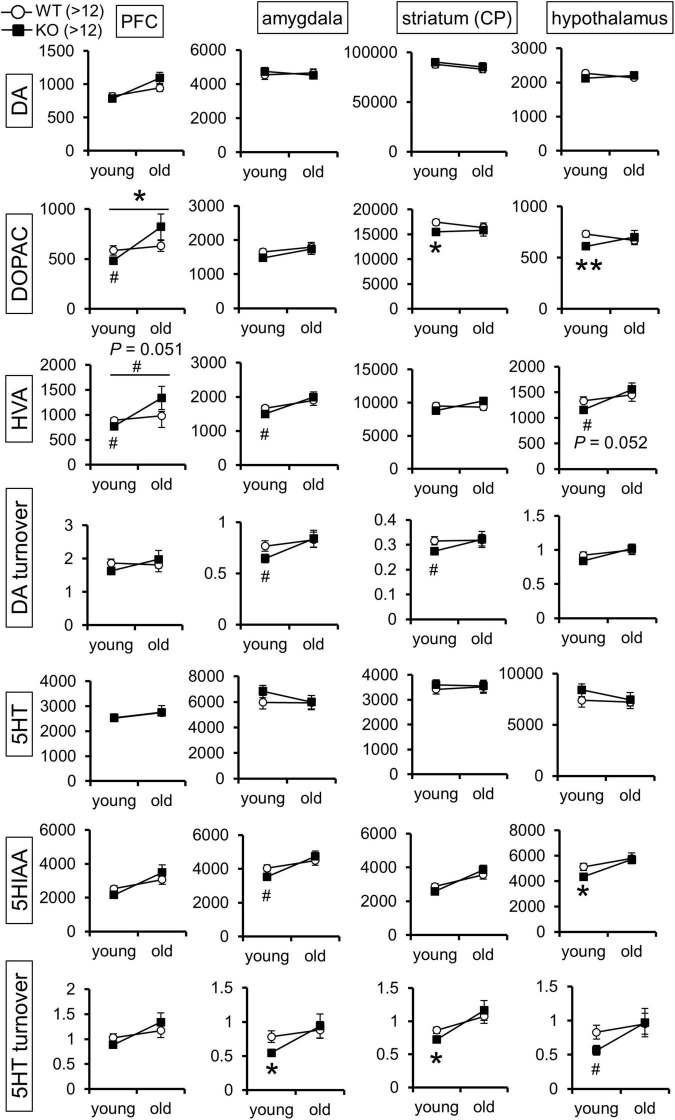
Monoamine content is altered in the brain of Lrtm2 KO mice. Graphs indicate mean monoamine concentration values (pg/mg protein; *white circle*, WT; *black square*, KO) for 5-month-old (young) and 13–20-month-old (old) mice, *n* = 13 (WT, young), 14 (KO, young), 13 (WT, old), or 13 (KO, old) mice for each group. *Error bars*, standard error of the mean (SEM). ^#^*p* < 0.1; *p < 0.05; **p < 0.01 in Student’s *t*-test. Top horizontal bars in PFC-DOPAC and PFC-HVA graphs indicate #, *p* < 0.1; *p < 0.05, two-way ANOVA (p_*genotype* × ag*e*_). HVA, homovanillic acid; 5HIAA, 5-hydroxy indole acetic acid. The results for hippocampus, noradrenaline, and its metabolites are indicated in Supplementary Figure 5.

The above results of the Lrtm2 expression analysis and behavioral tests led us to hypothesize that brain monoamine dynamics might be altered in Lrtm2 KO mice. We then measured the levels of DA, 5-HT, noradrenaline (NA), and their metabolites in the prefrontal cortex, hippocampus, amygdala, dorsal striatum (CP), and hypothalamus of the two age groups (Figure 6). As a result, there were significant differences in DA and 5-HT metabolites in multiple brain regions. For DA, its metabolite DOPAC showed a reduction in the dorsal striatum (*t* = 2.12, *p* = 0.043) and hypothalamus (*t* = 2.99, *p* = 0.0056) in the young group Lrtm2 KO and showed age-dependent changes (low in young and high in old Lrtm2 KO) in prefrontal cortex [*F*_(1_, _56)_ = 4.24, p_*genotype*×*age*_ = 0.044, two-way ANOVA]. Another DA metabolite, homovanillic acid (HVA), showed analogous changes but not significantly [*F*_(1_, _56)_ = 3.98, p_*genotype*×*age*_ = 0.051]. For 5-HT, its metabolite 5-hydroxy indole acetic acid (5HIAA) was decreased in the hypothalamus (*t* = 2.22, *p* = 0.034), and 5-HT turnover (5HIAA/5-HT) was decreased in both the amygdala (*t* = 25.6, *p* = 0.024, Welch’s *t*-test) and the dorsal striatum (*t* = 25.1, *p* = 0.016, Welch’s *t*-test) of the young group Lrtm2 KO mice. For NA, significant changes were limited to the hippocampus, where NA increased slightly in young Lrtm2 KO mice (*t* = 29, *p* = 0.022) (Supplementary Figure 5). Thus, monoamine content analysis revealed that Lrtm2 deficiency affects monoaminergic systems. In particular, we observed changes in the dopaminergic system, which may be associated with the Lrtm2 abundant striatal projections and behavioral abnormalities in Lrtm2 KO mice.

### Altered Presynaptic Protein Distribution in Leucine-Rich Repeats and Transmembrane Domains 2 Knockout Striatal Projections

**FIGURE 7:**
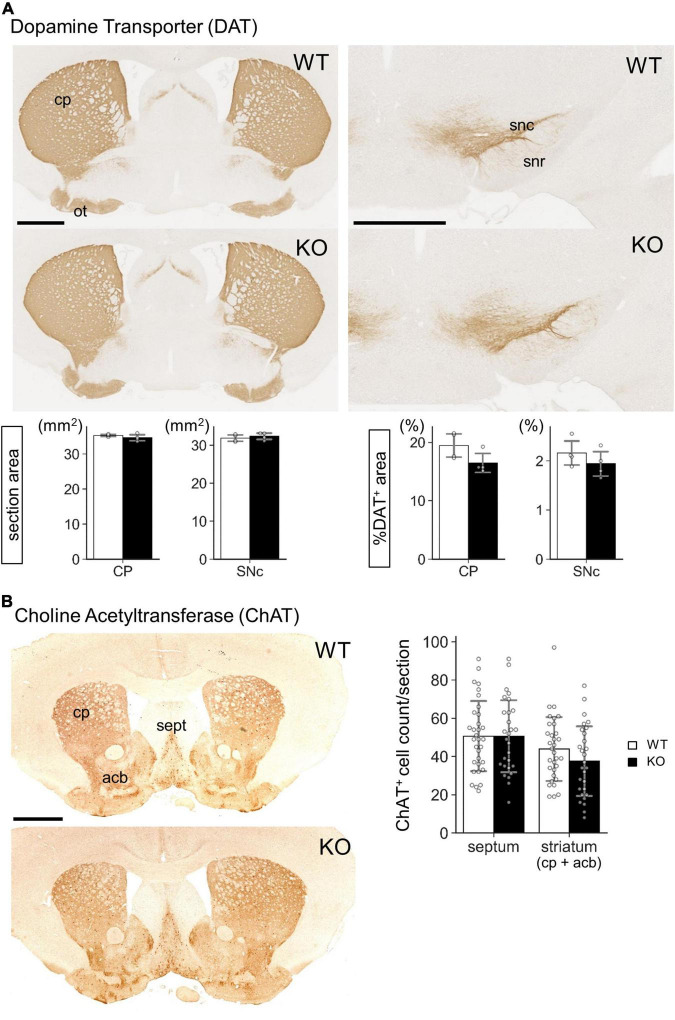
Gross morphology of Lrtm2 KO striatum and substantia nigra. Immunostaining of Lrtm2 WT and KO mouse brains using **(A)** anti-dopamine transporter (DAT) antibody or **(B)** anti-choline acetyl transferase (ChAT) antibody. **(A)** DAT immunostaining. *Left-graphs*, entire areas of coronal sections through CP or SNr. *Right-graphs*, areas of DAT-immunopositive areas are indicated as percentages to entire section areas. *n* = 4 mice for each genotype. Mean of 4 images was used for a mouse. **(B)** ChAT immunostaining. *Graph*, quantification of ChAT-immunopositive cell numbers in septum or in striatum. *n* = 32 or 28 sections from 4 mice for each genotype. *Error bars*, SD. There were not significant differences between WT and KO in either region. acb, nucleus accumbens; cp, caudoputamen; ot, olfactory tubercle; sept, septum; snc, substantia nigra pars compacta; snr, substantia nigra pars reticulata. *Scale bars*, 1 mm.

**FIGURE 8:**
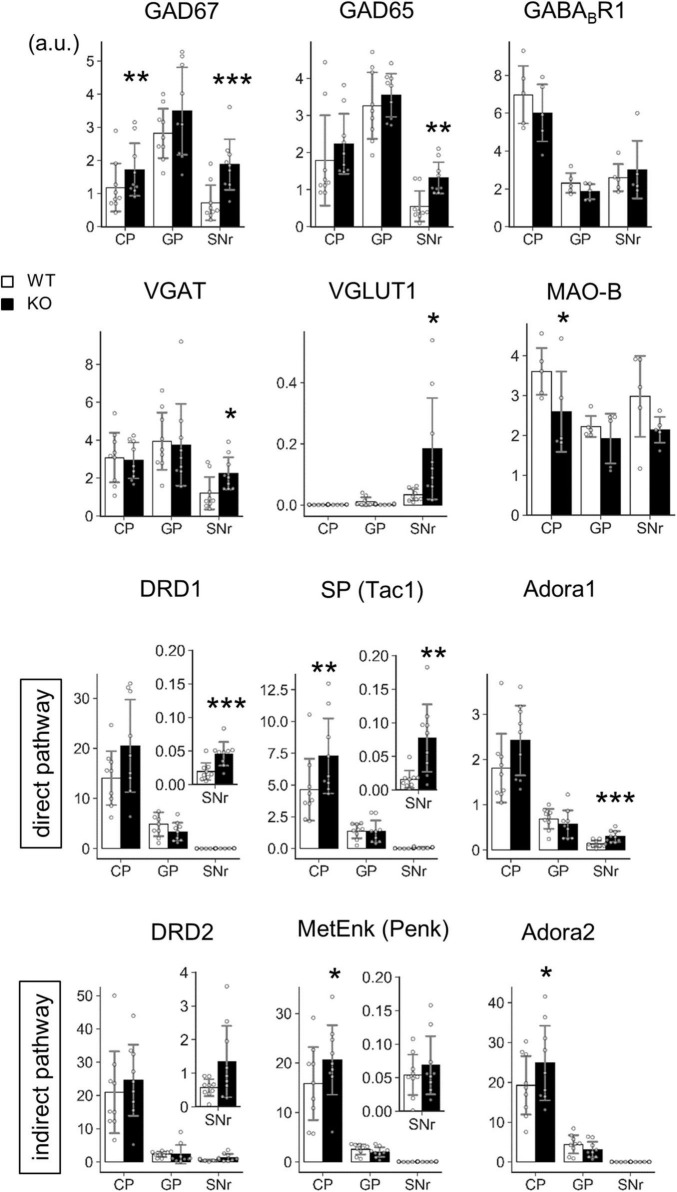
Transcript level analysis of Lrtm2 KO striatal projections. Transcript levels of the indicated proteins are determined by quantitative PCR analysis using frozen brain punches from the caudoputamen (CP), globus pallidus (GP), and SNr. 6–14 M-old mice (*n* = 9, 4 males and 5 females for each genotype) were analyzed. The mean of three GAPDH normalized RNA level values is used as a representative mRNA level value for a mouse. *Gray circles* indicate the individual values for each mouse. *p < 0.05; **p < 0.01; ***p < 0.001 in paired *t*-test. *Error bars*, SD. Scale, arbitrary unit. Relative transcript levels (WT = 100%) are indicated in Supplementary Figure 6.

**FIGURE 9:**
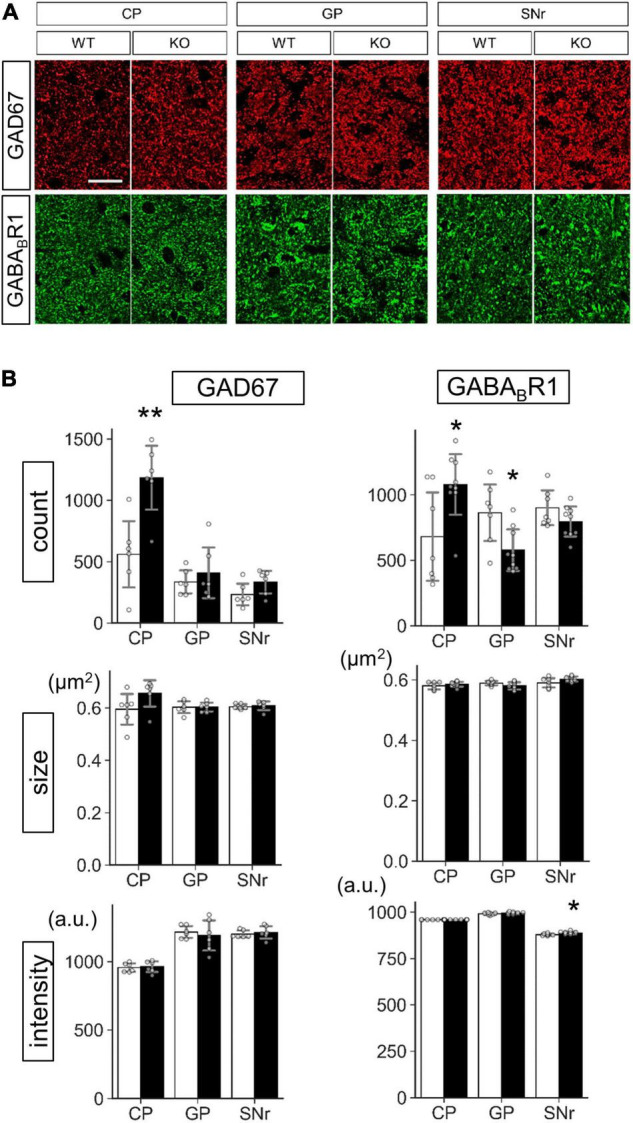
Protein level analysis of Lrtm2 KO striatal projections. Protein levels were estimated by quantifying punctate immunopositive signals from each region after immunofluorescence staining. **(A)** Representative images. Error bars, SD. Scale bar, 20 μm. **(B)** Mean of four sections is used as a representative value for a mouse. 10–12 week-old mice (*n* = 6, 3 males and 3 females) were analyzed. *Gray circles* indicate the individual values, which are the means of six sections per mouse for each mouse. *p < 0.05; **p < 0.01 in Student’s *t*-test. *Error bars*, SD. Additional results are indicated in Supplementary Figures 8–10.

Our next challenge was to clarify the molecular events that occurred in the Lrtm2 KO striatal projection system. The morphology of striatum or substantia nigra did not show clear differences between WT and KO mice, as shown by DA transporter or choline acetyltransferase immunostaining (Figure 7). We then performed a quantitative analysis for mRNA and/or proteins of genes that are expressed or functionally important in the striatal projection system (Figures 8, 9). These included proteins involved in inhibitory presynapse [GAD67 (Gad1), GAD65 (Gad2), VGAT (Slc32a1), GABA_*B*_R1 (Gabbr1)], excitatory presynapse [VGLUT1 (Slc17a7)], direct pathway SPN [DRD1 (Drd1), Substance P (SP, Tac1), Adora1 (Adora1)], indirect pathway SPN [DRD2 (Drd2), Met-enkephalin (MetEnk, Penk), Adora2 (Adora2)], and monoamine metabolizing enzyme [MAO-B (Maob)].

The mRNA levels of GAD67, GAD65, DRD1, SP, and Adora1 were commonly increased at SNr (GAD67, *t* = 5.86, *p* = 0.00038; GAD65, *t* = 3.91, *p* = 0.0045; DRD1, *t* = 6.18, *p* = 0.00027; SP, *t* = 3.52, *p* = 0.0078; Adora1, *t* = 5.83, *p* = 0.00039) (Figure 8). GAD67, SP, MetEnk, and Adora2 were increased (GAD67, *t* = 4.78, *p* = 0.0014; SP, *t* = 4.69, *p* = 0.0016; MetEnk, *t* = 2.93, *p* = 0.019; Adora2, *t* = 2.75, *p* = 0.025), and MAO-B levels were decreased (*t* = 3.77, *p* = 0.020) in the dorsal striatum (caudatoputamen, CP) (Figure 8). In terms of pathways, direct pathway genes (DRD1, SP, and Adora1) were all upregulated in KO SNr, whereas the indirect pathway genes (DRD2, MetEnk, and Adora2) did not show differences in GP or SNr (Figure 8). Collectively, transcript levels of several marker genes were affected by Lrtm2 deficiency either at CP or SNr.

Protein level analysis was performed by particle analysis of immunostaining signals (Figure 9 and Supplementary Figures 7–10). The number of GAD67 immunopositive particles increased (*t* = 3.72, *p* = 0.0040) in the Lrtm2 KO CP. The number of GABA_*B*_R1 particles also increased in CP (*t* = 2.61, *p* = 0.021), but decreased in GP (*t* = 2.84, *p* = 0.013) (Figure 9). GAD65 immunopositive signals did not show clear differences in any region (Supplementary Figures 8, 10). DRD1 showed a higher intensity in SNr (*t* = 3.18, *p* = 0.0099) (Supplementary Figures 9, 10), and MAO-B showed lower integrated density in SNr (*t* = 2.86, *p* = 0.017) (Supplementary Figure 7). The other proteins did not show significant differences between WT and KO mice (Supplementary Figures 8–10).

**FIGURE 10:**
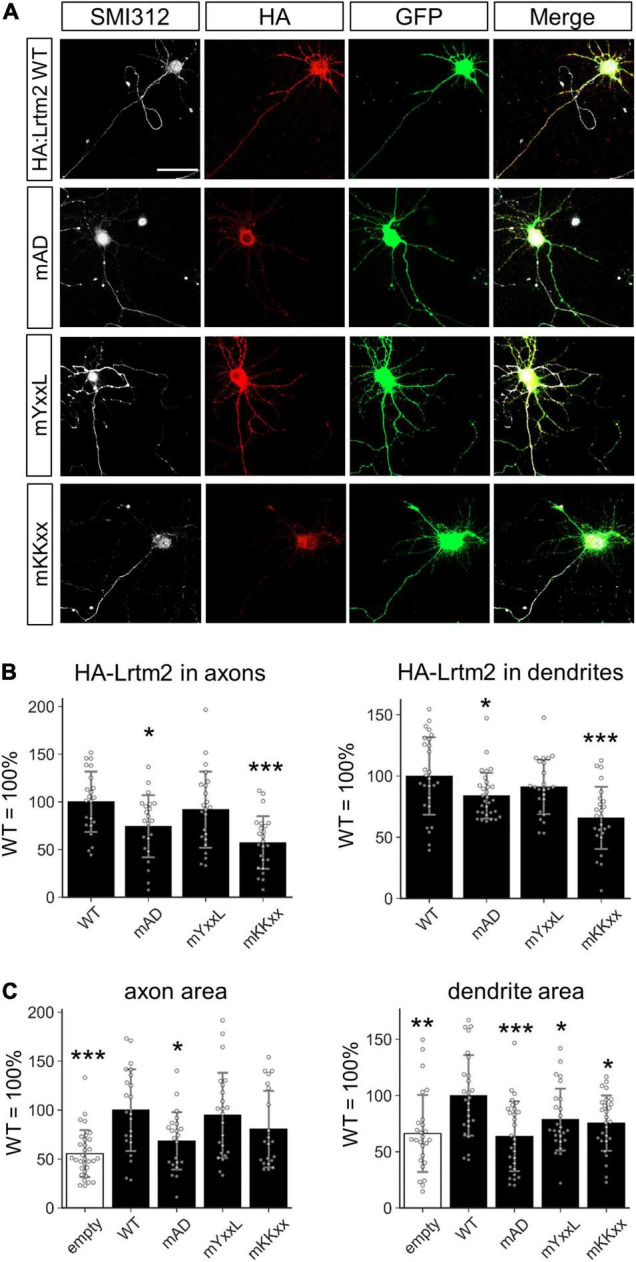
Motifs that define the subcellular localization of Lrtm2. **(A)** Representative images. HA-Lrtm2 WT and mutants are detected by anti-HA epitope tag antibody (*red*). Transfected cells are identified by membrane-anchored CFP (GFP, *green*). *Scale bar*, 50 μm. Representative images for MAP2 immunostaining are indicated in Supplementary Figure 11. **(B)** Quantification of HA-Lrtm2 WT or HA-Lrtm2 mutants in axons (*left*, defined as a ratio of HA-positive area to SMI312-positive area) or dendrites (*right*, defined as a ratio of HA-positive area to MAP2-positive area). *n* = 19–24 cells for each expression construct. Analysis of the neurites shapes is indicated in Supplementary Figure 12. **(C)** Effects of HA-Lrtm2 WT on neurites’ areas. *Gray circles* indicate the individual value of each cell. *p < 0.05; **p < 0.01; ***p < 0.001 in *p < 0.05; **p < 0.01; ***p < 0.001 in ANOVA and *post hoc* Dunnett test, compared to WT values. *Error bars*, SD.

Based on the results of mRNA and protein level analyses, our attention was attracted by the altered distribution of GAD67 and GABA_*B*_R1 proteins. Either protein dynamics might be affected by the absence of the Lrtm2 protein, which is densely distributed in the SNr. Therefore, we aimed to observe the effects of Lrtm2 protein mislocalization on GAD67 and GABA_*B*_R1 localization.

### Identification of Leucine-Rich Repeats and Transmembrane Domains 2 Protein Sorting Elements

To mislocalize the Lrtm2 protein, we had to verify the candidate protein sorting signals in the cytoplasmic region of Lrtm2 (Figure 2). For this purpose, the three candidate sequences in were mutated as follows: YxxL/Yxxφ (endocytosis-associated/somatodendritic targeting), YASL → AASA; KKxx (ER retrieving signal), KKRQ → AARQ; acidic domain (AD) (axon targeting), GDPEGEHED → GRPRGRHRR. After transfecting their expression vectors with an amino-terminal epitope (HA) tag, we examined the localization of mutant Lrtm2 proteins within cultured hippocampal neurons (Figure 10 and Supplementary Figures 11, 12). Mutated AD (mAD) or mutated KKxx (mKKxx) containing Lrtm2 showed poor localization in both axons and dendrites (mAD-axon, *p* = 0.038; mKKxx-axon, *p* = 0.00015; mAD-dendrite, *p* = 0.049; mKKxx-dendrite, *p* < 0.00001, ANOVA and *post hoc* Dunnett test) (Figure 10B). We also examined the neurites areas (Figure 10C) and the neurites shapes (Supplementary Figure 12) of the transfectants. HA-Lrtm2 increased the axon and dendrite areas (axon, *t* = 4.43, *p* = 0.00011, Welch’s *t*-test; dendrite, *t* = 3.40, *p* = 0.0013) (Figure 10C). Regarding mutants, the AD mutation reduced the axon/dendrite area-increasing ability (axon, *p* = 0.028; dendrite, *p* < 0.00001, ANOVA and *post hoc* Dunnett test) and both mYxxL and mKKxx mutations reduced dendrite area-increasing ability (mYxxL, *p* = 0.035; mKKxx, *p* = 0.012, ANOVA and *post hoc* Dunnett test) in comparison to HA-Lrtm2 wild-type (Figure 10C). In terms of neurites shapes, HA-Lrtm2 reduced the dendrite complexity (*p* = 0.00048, two-way repeated measures ANOVA and *post hoc* Dunnett test), and both mYxxL and mKKxx impaired the reducing ability (mKKxx, *p* = 0.00026; mYxxL, *p* = 0.013, two-way repeated measures ANOVA and *post hoc* Dunnett test) (Supplementary Figure 12). Taken together, both mKKxx and mAD impaired the neurites localization of HA-Lrtm2 while all of the three mutants affected the neurite modulating abilities of HA-Lrtm2 differentially.

### Leucine-Rich Repeats and Transmembrane Domains 2 Co-expression Enhanced Axon Localization of GAD67: GFP and GABA_*B*_R1: GFP in Cultured Neurons

**FIGURE 11:**
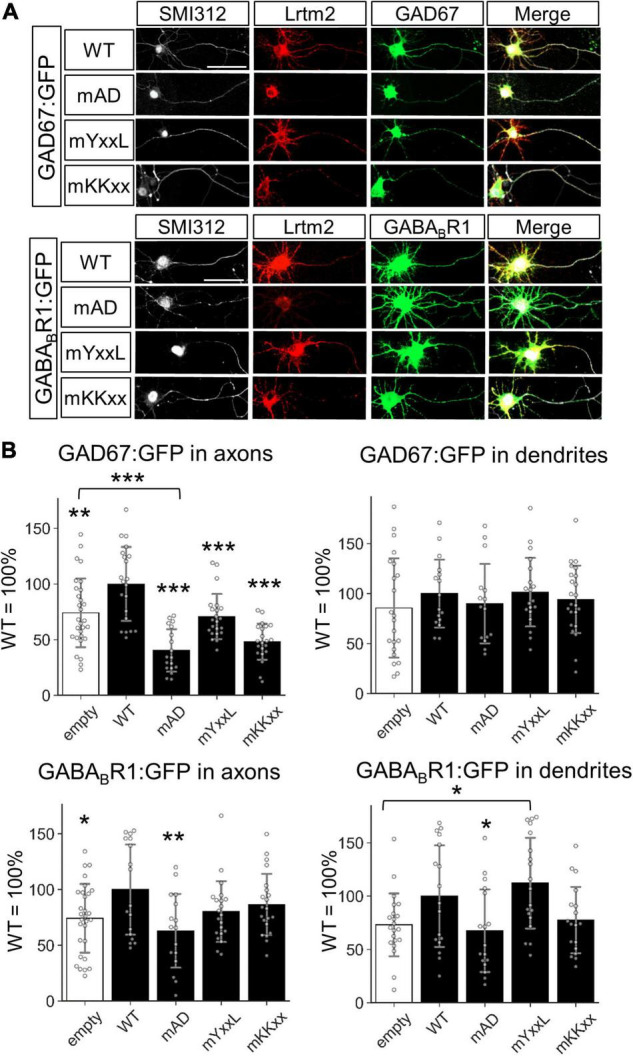
Lrtm2 overexpression enhances axon targeting of GAD67:GFP and GABA_*B*_R1:GFP. Cultured hippocampal neurons are transfected with empty, Lrtm2 or its mutant-expressing plasmid with the GAD67:GFP or GABA_*B*_R1:GFP expressing plasmid. **(A)** Representative images. **(B)** The distribution of the GFP fusion proteins in the axon (*left*, defined as a ratio of GFP-positive area to the SMI312-positive area) or dendrites (*right*, defined as a ratio of GFP-positive area to MAP2-positive area) is examined. *n* = 15–23 cells for each combination of the expression constructs. *Error bars*, SD. *Gray circles* indicate the individual value of each cell. *Scale bars*, 50 μm. Representative images for MAP2 immunostaining are indicated in Supplementary Figure 13. *p < 0.05; **p < 0.01; ***p < 0.001 in *p < 0.05; **p < 0.01; ***p < 0.001 in ANOVA and *post hoc* Dunnett test, compared to WT values unless otherwise indicated.

We then examined the effects of untagged Lrtm2, mAD, mYxxL, or mKKxx co-existence with the GFP-tagged GAD67 (GAD67:GFP) or GFP-tagged GABA_*B*_R1 (GABA_*B*_R1:GFP) (Figure 11 and Supplementary Figure 13). Regarding the axon localization of the GFP-tagged proteins, Lrtm2 enhanced axon localization of both GAD67:GFP (*t* = 2.78, *p* = 0.0077) and GABA_*B*_R1:GFP (*t* = 2.38, *p* = 0.022) (Figure 11). All three mutants showed reduced GAD67:GFP axon localization, and mAD reduced it more severely (mAD, *p* < 0.00001; mYxxL, *p* = 0.00039; mKKxx, *p* < 0.00001, ANOVA and *post hoc* Dunnett test), which was lower than the Lrtm2 absence (empty vector) control (*t* = 4.52, *p* = 0.000045, Welch’s *t*-test) (Figure 11). GABA_*B*_R1:GFP axon localization was reduced by mAD (*t* = 2.78, *p* = 0.0092, ANOVA and *post hoc* Dunnett test), but not by mYxxL or mKKxx (Figure 11). Neither Lrtm2 nor any mutants affected GAD67:GFP dendrite localization. mYxxL enhanced GABA_*B*_R1:GFP dendrite localization (*t* = 3.32, *p* = 0.0020), and Lrtm2 wild-type showed similar tendency (*t* = 1.81, *p* = 0.068, Welch’s *t*-test) in comparison to the empty vector control (Figure 11 and Supplementary Figure 13). These results indicate that Lrtm2 overexpression can alter the subcellular localization of GAD67:GFP and GABA_*B*_R1:GFP in cultured neurons, particularly the axon targeting of both proteins.

### Leucine-Rich Repeats and Transmembrane Domains 2 mAD Mutant Was Defective in Redistributing GABA_*B*_R1 in the Brain of Leucine-Rich Repeats and Transmembrane Domains 2 Knockout Mice

**FIGURE 12:**
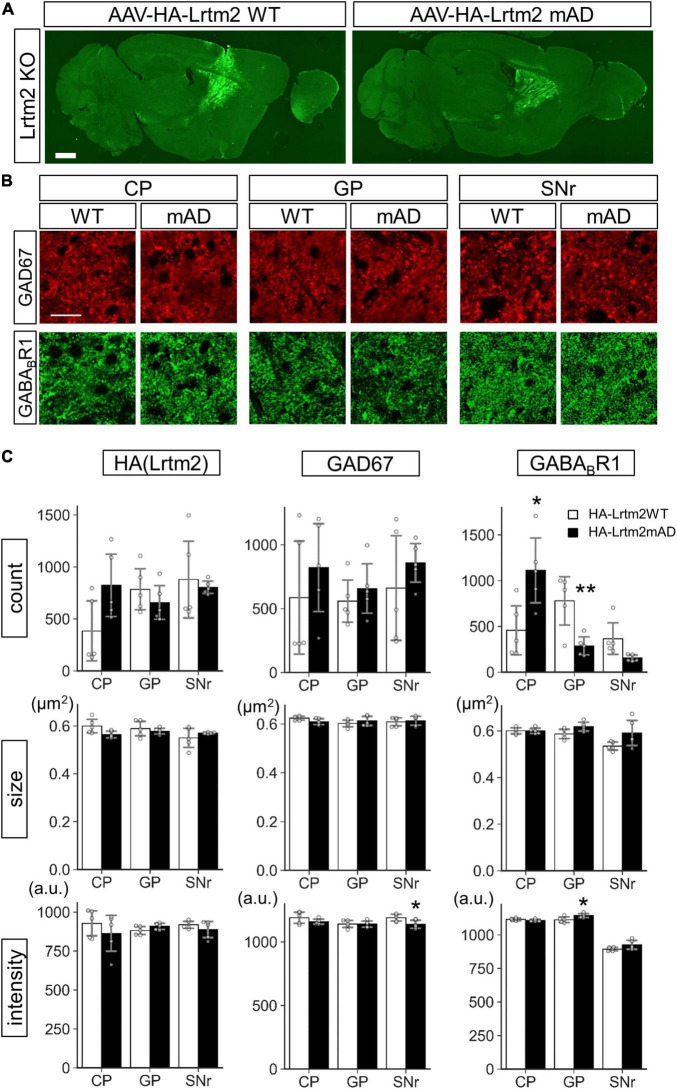
Different GAD67 and GABA_*B*_R1 sorting activities of HA-Lrtm2 WT and HA-Lrtm2mAD. HA-Lrtm2 WT or HA-Lrtm2mAD expressing AAV is injected into the right dorsal striatum of Lrtm2 KO mice (*n* = 5 mice, 5–27 M-old 2 males and 3 females for each construct). The injected mouse brains are subjected to immunostaining analysis using anti-GAD67 or anti-GABA_*B*_R1 antibodies. **(A)** Representative images. **(B)** Representative images at higher magnification. *Scale bars*, 1-mm-thick; 10-μm-thin. **(C)** Quantitative analysis. *Gray circles* indicate individual values, which are the means of two sections per mouse. *p < 0.05, **p < 0.01, Student’s -test. *Error bars*, SD.

We also examined the effects of HA-Lrtm2WT and HA-Lrtm2mAD expression in the brain. To this end, we injected AAVs that could express either protein into the Lrtm2 KO dorsal striatum (Figure 12). The size of HA-Lrtm2mAD immunoreactive particles tended to be smaller (*t* = 2.29, *p* = 0.052), but the number tended to be greater (*t* = 2.12, *p* = 0.067) than that of HA-Lrtm2WT in CP (Figures 12B,C), suggesting that the localization of Lrtm2 protein was affected by the mAD mutation also *in vivo*. The GABA_*B*_R1 signal count was higher in CP (*t* = 2.94, *p* = 0.018) but lower in GP (*t* = 3.50, *p* = 0.0081) and SNr (*t* = 2.41, *p* = 0.069, Welch’s *t*-test) compared to HA-Lrtm2mAD to HA-Lrtm2WT. These results suggested that HA-Lrtm2mAD was defective in enhancing axon sorting of endogenous GABA_*B*_R1, in agreement with the *in vitro* experiment using GABA_*B*_R1: GFP (Figure 11). Regarding the GAD67-derived signals, the intensity was lower in SNr in HA-Lrtm2mAD than in HA-Lrtm2WT (*t* = 2.49, *p* = 0.038) (Figure 12). Collectively, the localization of GAD67 and GABA_*B*_R1 was differentially affected between HA-Lrtm2WT and HA-Lrtm2mAD, supporting the role of Lrtm2 in protein sorting to the inhibitory axon terminals of SPN.

### GAD67: GFP Was Retained in the Leucine-Rich Repeats and Transmembrane Domains 2 Knockout Dorsal Striatum

**FIGURE 13:**
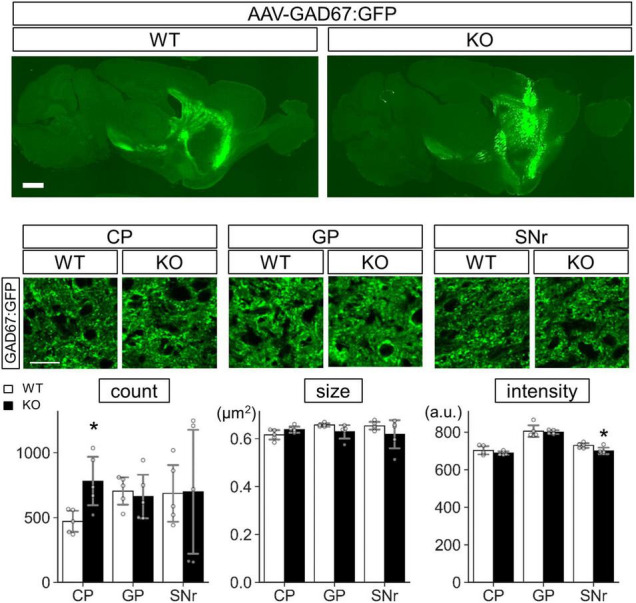
GAD67:GFP is stacked in the cell body of Lrtm2 KO SPNs. GAD67: GFP expressing AAV is injected into the right dorsal striatum of Lrtm2 WT or KO mice. The distribution of GAD67:GFP was examined 12 days after injection. *Top*, representative GFP signal distribution in Lrtm2 WT or Lrtm2 KO mice (*n* = 5, 4–25 M-old 1 male and 4 females for each genotype). *Scale bars*, 1-mm-thick; 10-μm-thin. *Bottom*. Quantification of GFP signals in CP, GP, and SNr. *Gray circles* indicate individual values, which are the means of two sections per mouse. *p < 0.05 in Student’s -test. *Error bars*, SD.

Concerning GAD67 in Lrtm2 KO SPN, increased GAD67 expression was thought to reflect increased mRNA levels, but the difference was not so clearly seen in SNr. To test whether exogenous GAD67 protein is affected by Lrtm2 deficiency, we expressed GAD67:GFP that can be transported into presynaptic clusters (Kanaani et al., 2010) in the dorsal striatum of Lrtm2 WT and KO mice by injecting AAV-CAG-GAD67:GFP into the hemi-lateral dorsal striatum. The GFP signals were widely detected in the dorsal striatum, but were scarce at SNr on 4 days after the injection (Supplementary Figure 14). We then quantified GFP signals at CP, GP, and SNr 10–12 days after the injection (Figure 13). As a result, the GFP signal count was higher in the KO dorsal striatum than in the WT dorsal striatum (*t* = 3.05, *p* = 0.016) as we observed for endogenous GAD67 protein. In addition, the GFP signal intensity in the KO SNr was lower than the WT SNr (*t* = 2.66, *p* = 0.029) (Figure 13). It was thought that the increased count in CP represents increased GAD67-GFP-positive Golgi membranes and cytosolic vesicles (Kanaani et al., 2010). The decreased intensity in SNr may reflect the reduced GAD67-GFP amount per presynaptic terminal. These results indicated that Lrtm2 is necessary for proper GAD67 protein levels in CP, and the exogenous GAD67:GFP was less efficiently sorted to SNr in the absence of Lrtm2.

## Discussion

This study dealt with an uncharacterized protein, Lrtm2. We determined its molecular properties and biological significance. Lrtm2 is a small glycosylated single membrane-spanning protein with an LRR domain in the extracellular or luminal region and three protein sorting motifs in the cytoplasmic region. Its distribution in the brain is enhanced at the axon terminal of neurons and is abundant in striatal projections, thalamocortical projections, corticospinal projections, hippocampal mossy fibers, and projections within the olfactory bulb. The prevalent and consistent feature of the protein distribution implies that Lrtm2 is involved in establishing common neuronal properties among projection neurons. In agreement with the immunostaining results, Lrtm2 is recovered at both plasma membrane and internal compartments like recycling endosomes or synaptic vesicles.

In phylogenetic terms, Lrtm2 is distributed in vertebrate clades, similar to known neural LRR-transmembrane proteins that play diverse and essential roles in the CNS. It is noteworthy that the mammalian paralog, Lrtm1 (Dolan et al., 2007), shows a highly restricted expression in midbrain DA neuron progenitors and is utilized as a cell surface marker to enrich DA progenitors from stem cell-derived immature neurons (Samata et al., 2016). Together with the abundant distribution of Lrtm2 in striatonigral projections, we can conclude that the Lrtm family is involved in striatonigral neural circuits in both directions.

The role of Lrtm2 in higher brain function may be the control of voluntary movement initiation, motor function and learning, and exploratory behaviors, as indicated by Lrtm2 KO behavior analysis. The altered brain function is believed to reflect the altered function of many Lrtm2-expressing neural circuits. We speculate that striatonigral projections may be principally associated with behavioral abnormalities in Lrtm2 KO mice. This is because striatal projection systems are known to be involved in action selection (Gerfen and Surmeier, 2011) or in controlling goal-directed actions (Zhai et al., 2018), and altered monoamine dynamics, including decreased DA metabolite content and decreased serotonin turnover, were found in the dorsal striatum of Lrtm2 KO mice. In addition to striatonigral projections, alterations in corticospinal projection and thalamocortical projection properties are possible, considering their known roles in motor control (Ebbesen and Brecht, 2017) and attention and executive control (Halassa and Sherman, 2019).

In the monoamine content analysis, the following changes in DA or serotonin metabolism seem to be associated with Lrtm2 KO behavioral abnormalities. First, the administration of DA reuptake inhibitors is known to increase motor learning performance in the rotarod test (Shiotsuki et al., 2010). Second, mouse exploratory behavior in the open field is highly sensitive to striatal DA efflux (Patel et al., 2012). Third, altered serotonin signaling affects immobility in tail suspension tests, as evidenced by many studies that adopted pharmacological treatment or genetic manipulation in mice (Cryan et al., 2005). Thus, Lrtm2 expression, behavioral and neurochemical abnormalities in Lrtm2 KO mice, and the known roles of neural circuits and monoamine transmitters led us to focus on the role of Lrtm2 in SPN in this study.

Molecular marker analysis of SPN showed that both the striatal GAD67 protein (Figure 9) and exogenous protein (Figure 13, GAD67:GFP) were abnormally accumulated in Lrtm2 KO CP. These results suggest that Lrtm2 decreases CP GAD67 levels directly or indirectly. Because CP includes the somatodendritic compartment of SPN, the results were thought to reflect excessive GAD67 production in the soma/dendrites of SPN or the blockage of axonal transport of GAD67. In our *in vitro* experiments, all Lrtm2 mutants impaired axonal localization, but not in the dendritic location of GAD67: GFP (Figure 11), raising the possibility that Lrtm2 is involved in the axonal transport of GAD67. However, in SNr, GAD67:GFP, which was virally introduced into the dorsal striatum (Figure 13) and endogenous GAD67 protein (Figure 9), were comparable between WT and KO mice. Combining all results, in SPN, axon transport of GAD67 SPN may not require Lrtm2 *in vivo*, but Lrtm2 can control GAD67 axon transport *in vitro*. Therefore, we speculate that Lrtm2 is involved in GAD67 axon transport in a context-dependent or functionally redundant manner.

Meanwhile, it is probable that the increase in CP GAD67 in Lrtm2 KO mice reflects the increase in the level of GAD67 mRNA. GAD67 expression is regulated in a neuronal activity-dependent manner (Lau and Murthy, 2012). A voltage-gated sodium channel blocker, tetrodotoxin, reduces whereas a GABAA receptor blocker, picrotoxin, increases GAD67 expression in cultured hippocampal neurons at the transcription level (Lau and Murthy, 2012). Ablation of olfactory sensory neurons reduces GAD67 expression in the olfactory bulb (Lau and Murthy, 2012). Therefore, it is likely that Lrtm2 deficiency increases the excitability of SPN. It is important to determine whether any Lrtm2-binding proteins can influence neuronal excitability. Assuming that the increase in mRNA primarily causes GAD67 protein accumulation in CP, we must presume a restrictive or turning point for GAD67 axon transport. Accordingly, a previous study revealed that the proximal axons, including the axon initial segment (AIS), selectively sorts cargo entry. AIS is a scaffolding protein, ankyrin G, and an F-actin-dependent protein network (Song et al., 2009). However, little is known about the role of AIS in GAD67 axon entry. Meanwhile, GAD67 and its paralog GAD65 have been studied regarding the mechanism of localization in neurons (Kanaani et al., 1999; Fenalti et al., 2007; reviewed in reviewed in Wei and Wu, 2008; Kanaani et al., 2010). GAD67 is constitutively active and produces > 90% of GABA in the CNS, and GAD65 is transiently activated (Kanaani et al., 2010). The membrane anchoring and trafficking of GAD65 are mediated by intrinsic hydrophobic modifications; GAD67 remains hydrophilic yet is targeted to vesicular membrane pathways and synaptic clusters in neurons by both a GAD65-dependent (forming a heterodimer with GAD65) and a distinct GAD65-independent mechanism (Kanaani et al., 2010). It is possible that GAD65 acts as a rate-limiting factor for GAD67 axon transport and that Lrtm2 are associated with the presumptive GAD65 independent mechanism of GAD67 axon transport.

In contrast to GAD67, GABA_*B*_R1 protein particles were increased in KO CP, while GABA_*B*_R1 mRNA levels were comparable between WT and KO mice. Together with decreased particles in GP and increased intensity in SNr, Lrtm2 deficiency appears to have affected the subcellular localization of GABA_*B*_R1. Furthermore, the involvement of Lrtm2 in controlling GABA_*B*_R1 localization was supported by Lrtm2 WT or sorting signal-mutant overexpression *in vitro* and *in vivo*. Therefore, in the case of GABA_*B*_R1, Lrtm2 affected its protein distribution in an experimental design-independent manner, raising the possibility that GABA_*B*_R1 localization is under the control of Lrtm2 in physiological contexts. In previous studies, the N-terminal Sushi domain of GABA_*B*_R1 contains axon targeting signals (Biermann et al., 2010), Sushi domains interact with β-APP (Schwenk et al., 2016), and APP associates with c-Jun N-terminal kinase-interacting protein and calsyntenin proteins that link the APP/GABA_*B*_R1 complex in cargo vesicles to the axonal trafficking motor (Dinamarca et al., 2019). Whether or not Lrtm2 molecular function is linked to the known mechanism of GABA_*B*_R1 protein sorting remains an important challenge.

The altered protein localization of GAD67 and GABA_*B*_R1 may partly account for the behavioral abnormalities in Lrtm2 KO mice because previous studies showed their critical roles in SPN. Absence of GAD67 in direct pathway SPN impairs rotarod test performance (Heusner et al., 2008; Zhang et al., 2015). In unilateral 6-OHDA injection-induced hemiparkinsonian mice, the absence of GAD67 in direct pathway SPN does not cause abnormal involuntary movement (Zhang et al., 2015). In the hemiparkinsonian mice, tonic presynaptic inhibition by GABA_*B*_ receptors is lost, which potentiates evoked GABA release from SPN efferents to the SNr and drives motor sensitization (Borgkvist et al., 2015).

On the other hand, the effects of GAD67 and GABA_*B*_R1 disturbance on the modification of monoaminergic system in Lrtm2 KO remain unclear. Concerning the basis of the monoamine dysregulation, the reduced MAO-B mRNA at CP and the reduced MAO-B protein at SNr in Lrtm2 KO mice may reflect some adaptive responses of SPN to the Lrtm2 deficiency. It is known that MAO-B in several brain regions increases during aging of WT mouse (Saura et al., 1994; Mahy et al., 2000). Considering the age-dependent change of the monoamine metabolite, we hypothesize that Lrtm2 deficiency causes sustained alteration of the striatonigral outputs, which affects monoamine-fine-tuning mechanism including the control of MAO-B expression.

Finally, we describe the molecular properties, expression, and physiological roles of Lrtm2. Although this study is directed by the Lrtm2 protein distribution and Lrtm2 KO behavioral abnormalities, we cannot exclude its involvement in the trans-synaptic adhesion as are the case in other neural LRR-domain containing transmembrane proteins (Ko, 2012; Schroeder and de Wit, 2018). Whether Lrtm2 can behave as a trans-synaptic adhesion molecule depends on future studies. The behavioral and neurochemical phenotypes of Lrtm2 KO mice suggested that Lrtm2 is a significant molecule for further basic and clinical investigations. Although this study has chosen a limited number of molecular markers to explore Lrtm2 molecular functions, more comprehensive approaches are needed to fully describe the molecular consequences of Lrtm2 deficiency. The identification of a direct binding partner for Lrtm2 is essential to clarify its molecular function. Considering the preferential axon localization in several types of projection neurons, Lrtm2 may play a universal role associated with integrity of axon properties in these neurons. Therefore, we expect that further clarification of the Lrtm2-associated mechanism of SPN regulation would contribute to a better understanding of neuronal cell physiology.

## Data Availability Statement

The original contributions presented in the study are included in the article/Supplementary Material, further inquiries can be directed to the corresponding author/s.

## Ethics Statement

The animal study was reviewed and approved by the Animal Experiment Committee of RIKEN and Animal Care and Use Committee of Nagasaki University.

## Author Contributions

MI, KS, K-IK, NM, HO, and JA: conceptualization. MI, KS, K-IK, NM, KY, HM, MH, and JA: investigation, MI, KS, K-IK, and JA: writing–original draft. MI, KS, NM, KY, HO, and JA: writing–review and editing. MI and JA: funding acquisition. JA: supervision. All authors contributed to the article and approved the submitted version.

## Conflict of Interest

The authors declare that the research was conducted in the absence of any commercial or financial relationships that could be construed as a potential conflict of interest.

## Publisher’s Note

All claims expressed in this article are solely those of the authors and do not necessarily represent those of their affiliated organizations, or those of the publisher, the editors and the reviewers. Any product that may be evaluated in this article, or claim that may be made by its manufacturer, is not guaranteed or endorsed by the publisher.

## Footnotes

1
https://blast.ncbi.nlm.nih.gov/

2
https://pubmed.ncbi.nlm.nih.gov/

3
https://asia.ensembl.org/

4
https://www.expasy.org/

5
https://services.healthtech.dtu.dk/

6
http://n2t.net/addgene:59462
