# Supplementary material for: Leucine-Rich Repeats and Transmembrane Domain 2 Controls Protein Sorting in the Striatal Projection System and Its Deficiency Causes Disturbances in Motor Responses and Monoamine Dynamics
Source: Front Mol Neurosci. 2022 May 9;15:856315. doi: 10.3389/fnmol.2022.856315 (PMC9126195; doi:10.3389/fnmol.2022.856315)
Supplement: Supplementary file 1 [file Data_Sheet_1.pdf]

application	antibody name	antibody type	clone	RRID	Cat.#	manufacturer	host	dilution
immunoblot	anti-Lrtm2 antibody	polyclonal		NA	NA	this study	rabbit	1:1000
	anti-PSD-95 MAGUK scaffolding protein antibody	monoclonal	K28/43	AB_2292909	75-028	NeuroMab	mouse	1:20000
	anti-Rat Synaptophysin antibody	monoclonal	SVP-38	AB_477523	S 5768	Sigma-Aldrich	mouse	1:50000
immunostaining -cultured cells	anti-Lrtm2 antibody	polyclonal		NA	NA	this study	rabbit	1:10000
	anti-GFP antibody	polyclonal		AB_2534023	A10262	Invitrogen	chicken	1:1000
	anti-Neurofilament Marker (pan axonal, cocktail) antibody	monoclonal	SMI-312	AB_2566782	837904	BioLegend	mouse	1:500
	anti-MAP2 (2a / 2b) antibody	monoclonal	AP-20	AB_477171	M1406	Sigma-Aldrich	mouse	1:500
	anti-HA High Affinity	monoclonal	3F10	AB_390918	11867423001	Roche	rat	1:4000
immunostaining -tissue sections	anti-Lrtm2 antibody	polyclonal		NA	NA	this study	rabbit	1:2500
	anti-GFP antibody	polyclonal		AB_2534023	A10262	Invitrogen	chicken	1:500
	anti-HA High Affinity	monoclonal	3F10	AB_390918	11867423001	Roche	rat	1:4000
	anti-GAD67 antibody	monoclonal	1G10.2	AB_2278725	MAB5406	Millipore	mouse	1:1000
	anti-GAD65 antibody	monoclonal	GAD-6	AB_11214081	MAB351	Millipore	mouse	1:1000
	anti-GABA B Receptor 1 antibody	monoclonal		AB_941703	ab55051	Abcam	mouse	1:500
	anti-VGLUT1 antibody	monoclonal	N28/9	AB_2187693	75-066	NeuroMab	rabbit	1:1000
	anti-VGAT antibody	monoclonal	117G4	AB_887872	131 011	Synaptic Systems	mouse	1:250
	anti-Dopamine D1R/DRD1 antibody	monoclonal	SG2-D1a	AB_2277517	NB110-60017	Novus Biologicals	mouse	1:1000
	anti-Dopamine D2S/L receptor antibody	monoclonal	N186/29	AB_2094978	73-230	NeuroMab	mouse	1:10
	anti-Substance P antibody	polyclonal		NA	ab216412	Abcam	rabbit	1:300
	anti-Methionine Enkephalin antibody	polyclonal		AB_572250	20065	Immunostar	rabbit	1:300
	anti-MAO-B antibody	monoclonal	D-6	AB_2819030	sc-515354	Santa Cruz Biotechnology	mouse	1:300
	anti-Dopamine Transporter antibody	monoclonal	DAT-Nt	AB_2190413	MAB369	Millipore	rat	1:1000
	anti-Choline Acetyltransferase antibody	polyclonal		AB_2079751	ab144p	Millipore	goat	1:300

Supplementary Table 1. List of antibodies.

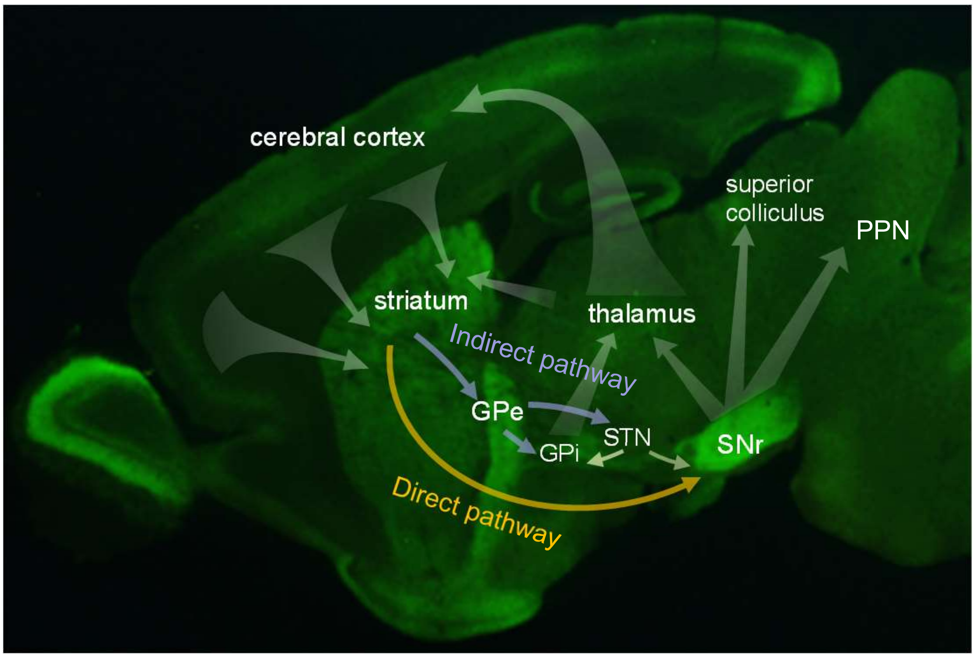

Supplementary Figure 1. Diagram of basal ganglia circuit. The image derives from an immunostaining for Lrtm2 (Figure 2F) and the circuit illustration is based on that in Gerfen and Surmeier (2001). GPe, external segment of globus pallidus; GPi, internal segment of globus pallidus; PPN, pedunculopointine nucleus; SNr, substantia nigra pars reticulata; STN, subthalamic nucleus. Note that Lrtm2 in striatal projection neurons is strongly detected at both their direct pathway target (SNr) and their indirect pathway target (GPe).

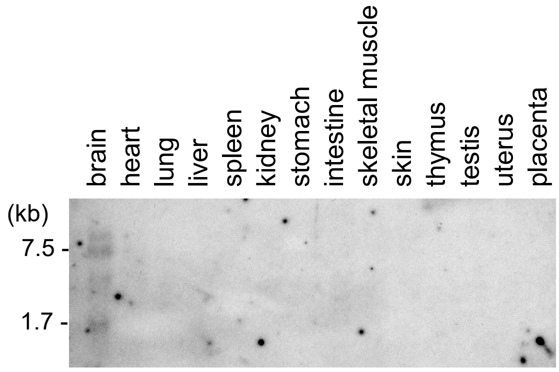

Supplementary Figure 2. Northern blot of Lrtm1.

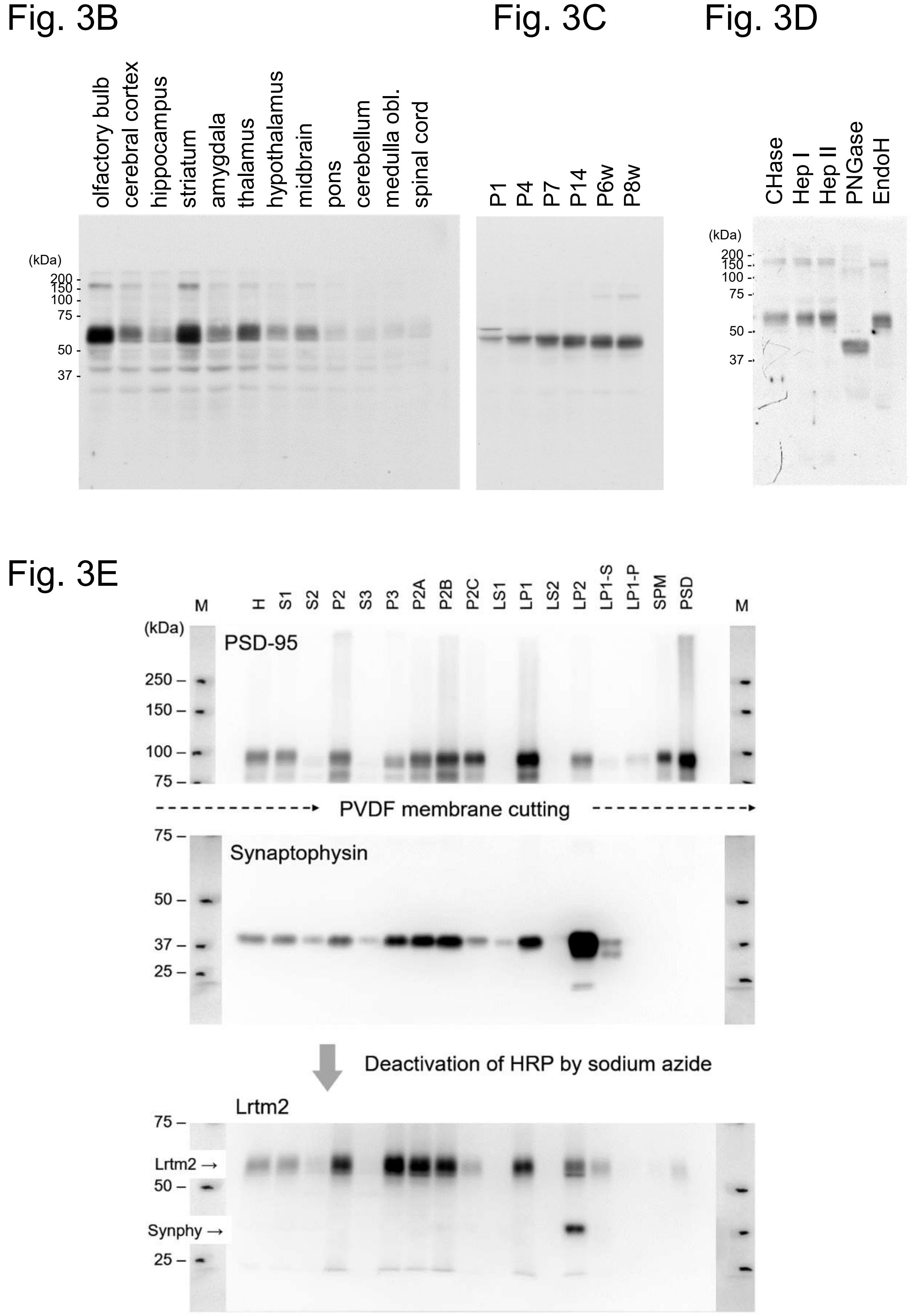

Supplementary Figure 3. Full immunoblots.

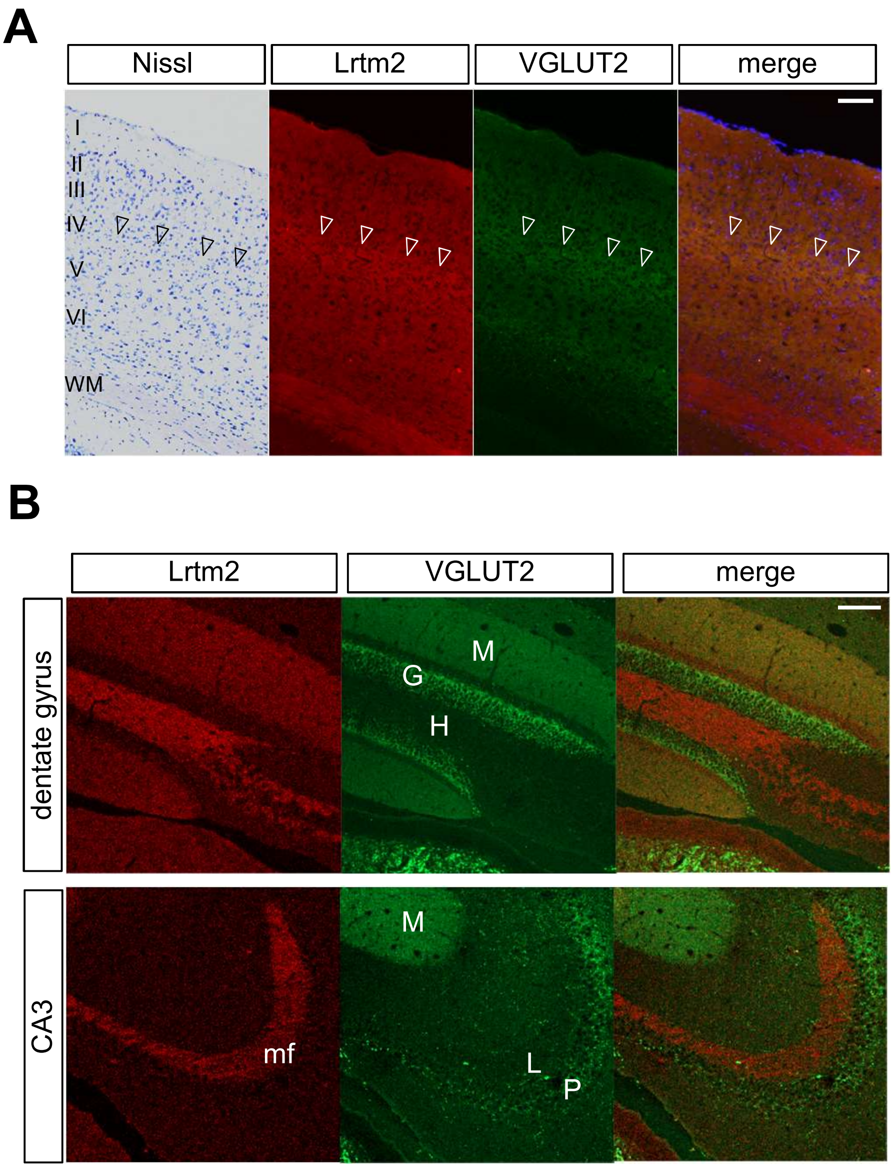

Supplementary Figure 4. Lrtm2 distribution in cerebral cortex and hippocampus. A coronal section (Bregma -2.46 mm) from an adult female WT mouse was immunostained for Lrtm2 (*red*) and VGLUT2 (*green*) with DAPI staining (*blue*). (A) High magnification views of primary visual cortex. From left to right, images for Nissl staining, Lrtm2 immunostaining, VGLUT2 immunostaining, and merged views. VGLUT2 is known to be preferentially located at layer 4 of the cerebral cortex (Fremeau et al., Neuron, 31, 247-260, 2001) (*arrowheads*). I-VI, the layer number of cerebral cortex, WM, white matter. (B) High magnification views of hippocampal dentate gyrus (top) and CA3 (bottom). G, stratum granulosum; H, hilus; L, stratum lucidum; M, stratum moleculare; mf, mossy fiber; P, stratum pyramidale. *Scale bars*, 100 μm.

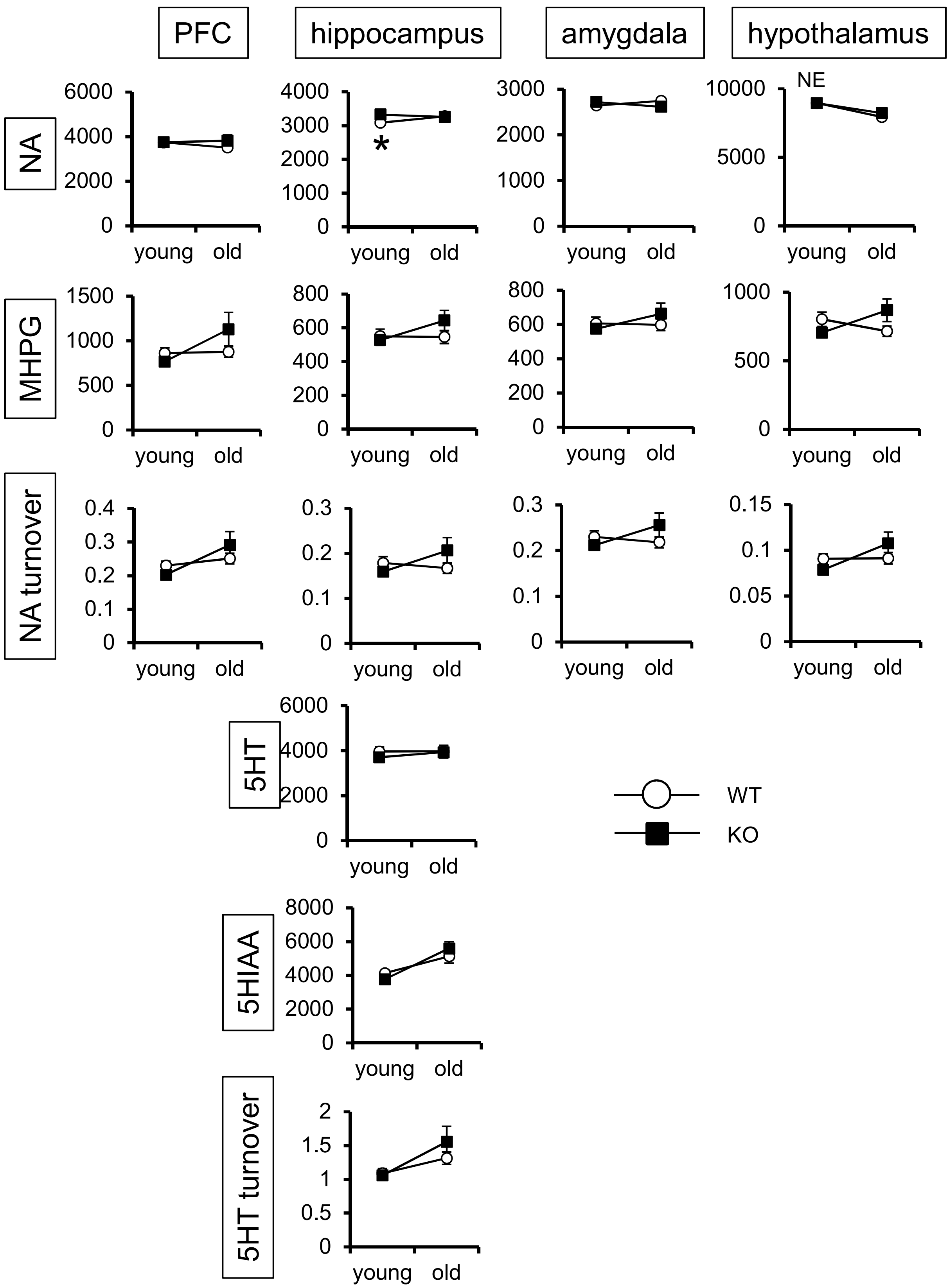

Supplementary Figure 5. Noradrenaline, 5-HT and their metabolites levels in Lrtm2 KO brain regions. In striatum, NA and MHPG were below the detection limit. Supplementary results for Fig. 6. Units are pg/(mg protein) except turnover ratio. mRNA amounts are indicated as those of whole brains are equal to 1.

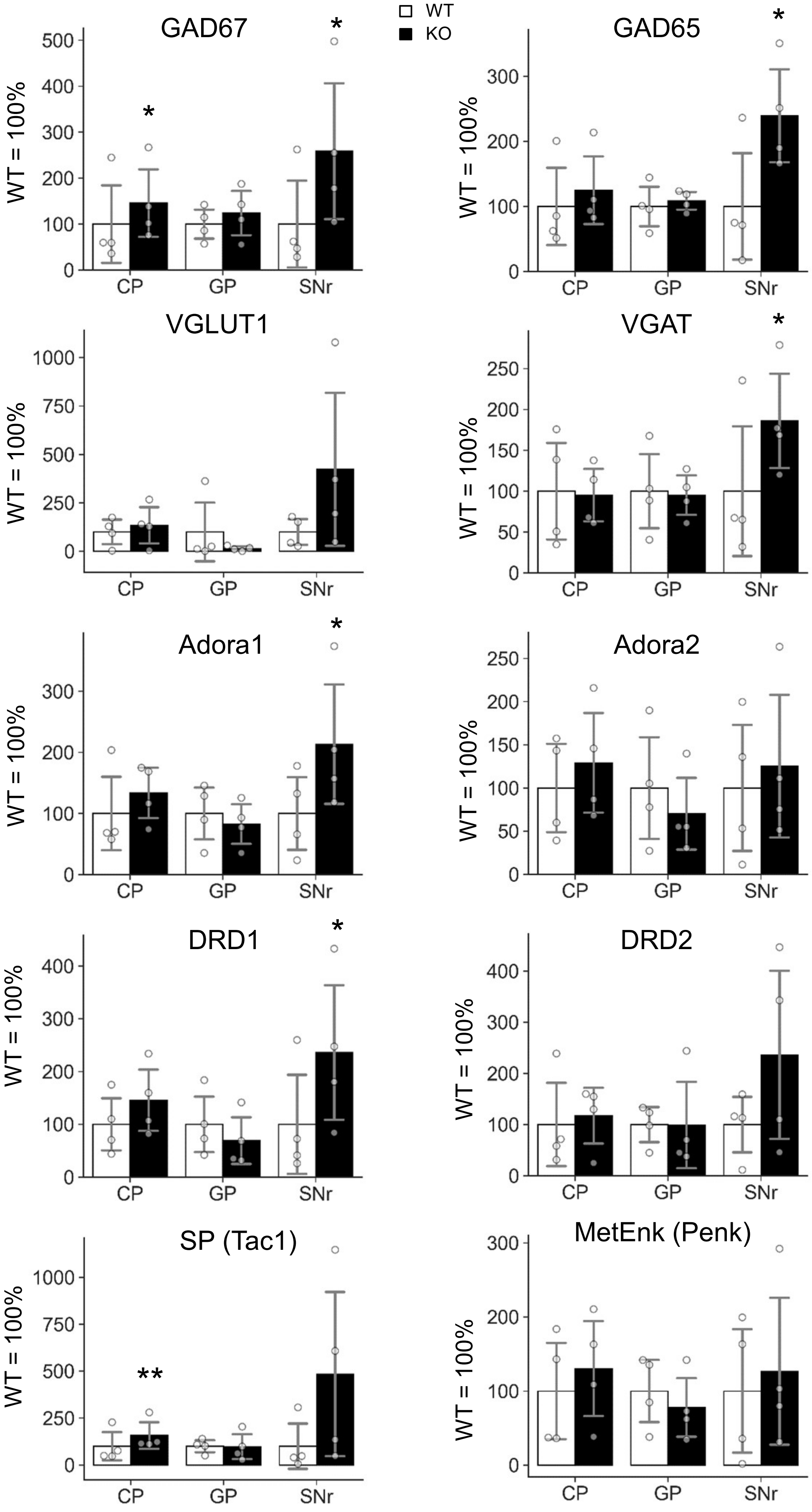

Supplementary Figure 6. Relative transcript amounts. mRNA amounts are indicated as those of whole brains are equal to 100%. *Error bar*, SD. *, $p < 0.05$; **, $p < 0.01$ in *t*-test.

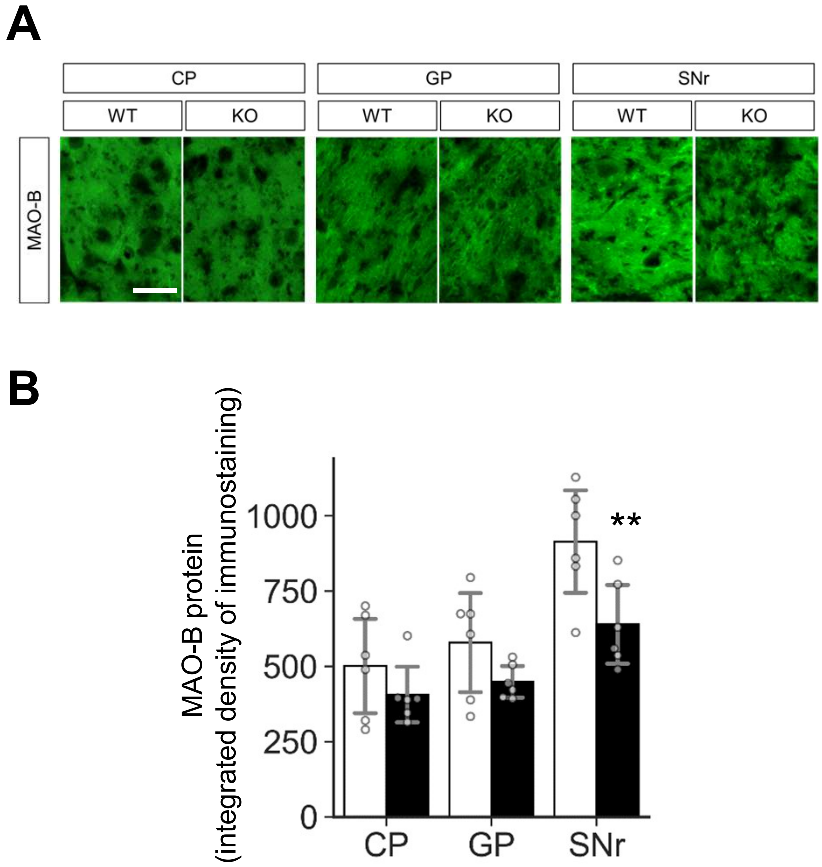

Supplementary Figure 7. MAO-B protein levels in Lrtm2 KO mice. Immunopositive signals in MAO-B immunostaining were quantified. *Error bar*, SD. *Scale bar*, 20 µm. *, $p < 0.05$; **, $p < 0.01$ in *t*-test.

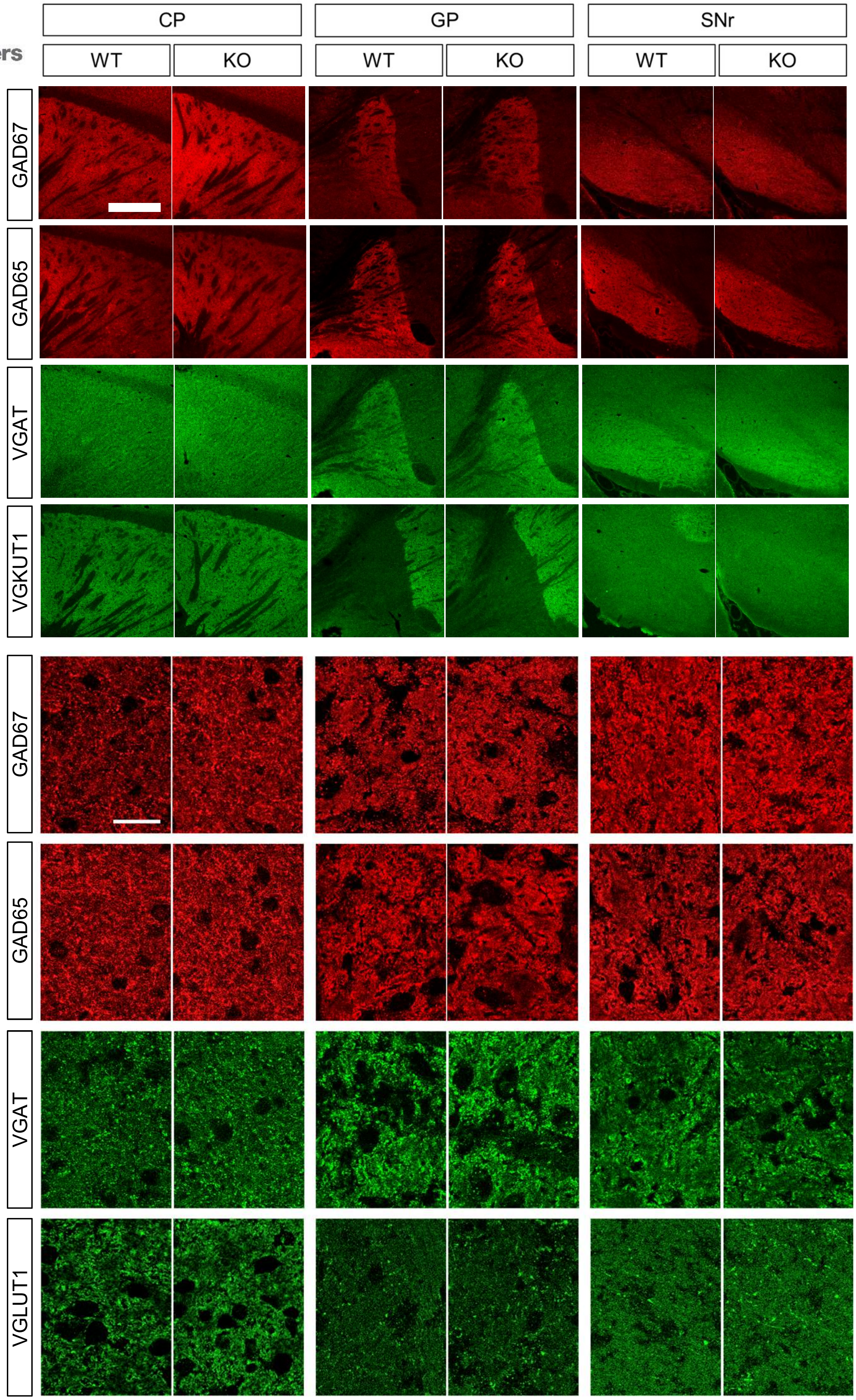

Supplementary Figure 8. Representative immunostaining images. Left indicated antibodies were used for Lrtm2 WT and KO brain parasagittal sections. Higher magnifications of caudopatamen (CP), Globus pallidus (GP), and Substantia nigra (SNr) are shown. *Scale bars*, 500-µm-thick, 20-µm-thin.

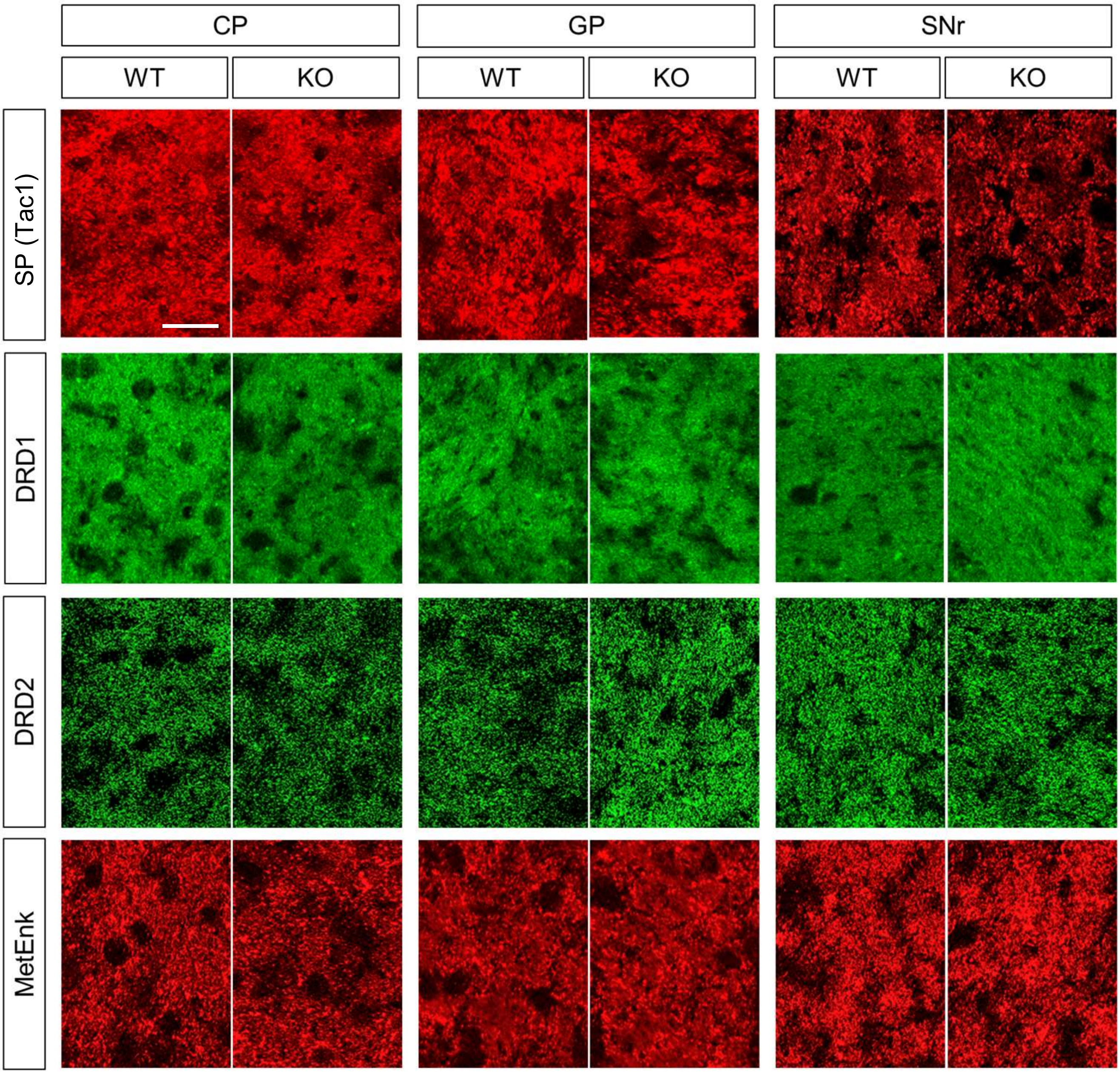

Supplementary Figure 9. Representative immunostaining images. Left indicated antibodies were used for Lrtm2 WT and KO brain parasagittal sections. Higher magnifications of caudopatamen (CP), Globus pallidus (GP), and Substantia nigra (SNr) are shown. *Scale bar*, 20 µm.

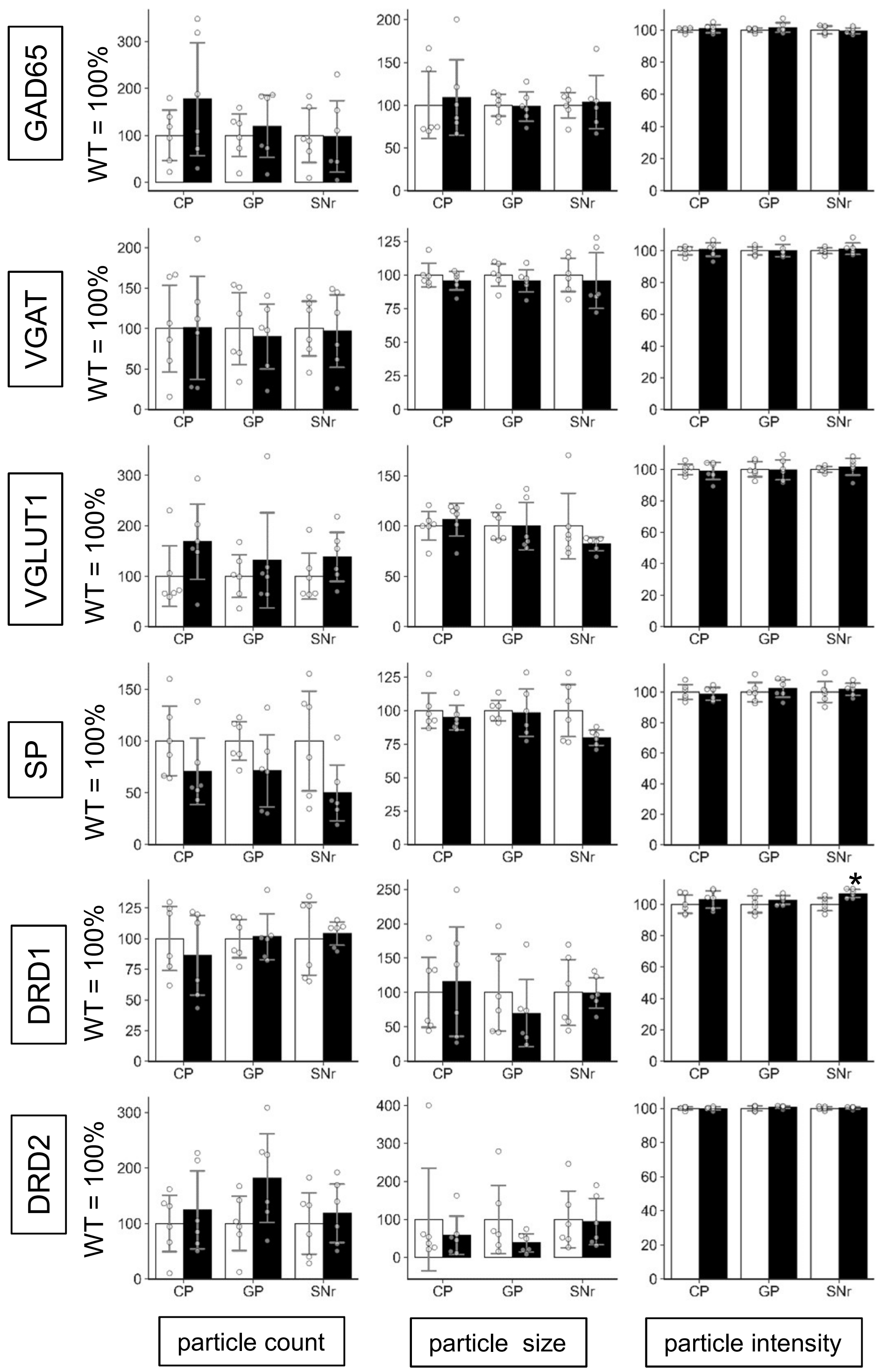

Supplementary Figure 10. Regional differences of the immunopositive signals. The values are normalized to those of WT to see the differences among the regions. *Error bar*, SD. *, $p < 0.05$ in *t*-test.

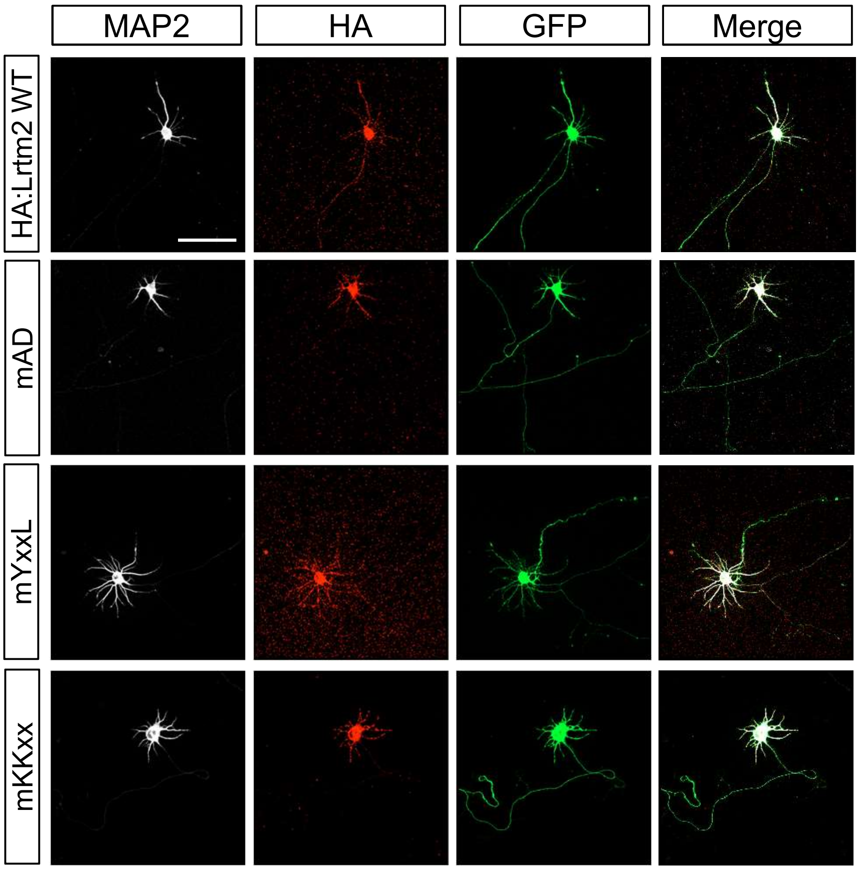

Supplementary Figure 11. Representative images for MAP2 and HA double immunostaining . MAP2-positive areas are shown in *white* in the left column. HA-Lrtm2 WT and mutants are detected by anti-HA epitope tag antibody (*red*). Transfected cells are identified by membrane-anchored CFP (GFP, *green*). Scale bar, 100 µm.

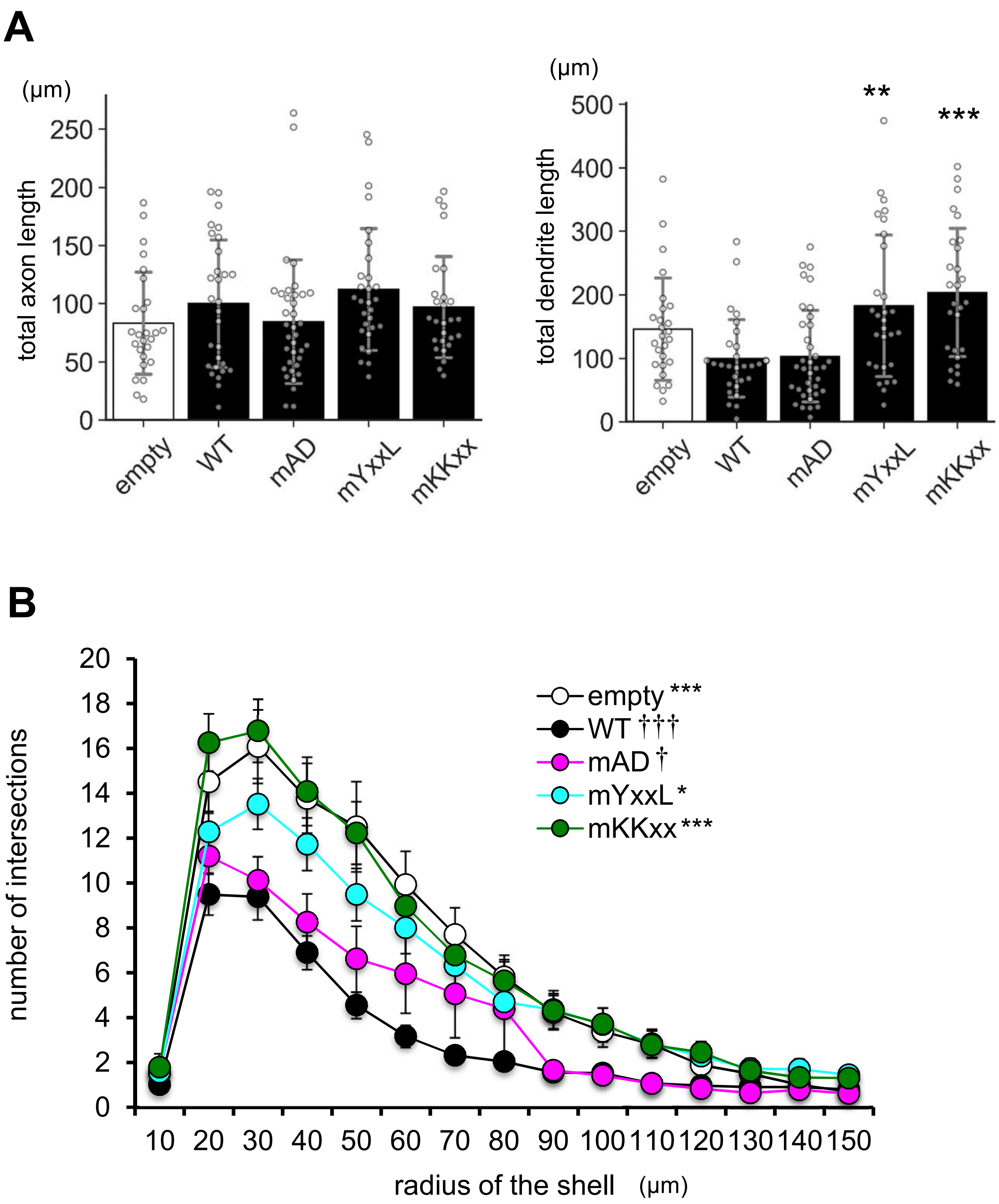

Supplementary Figure 12.
Quantification of the total nerites length and dendrite complexity of the hippocampal neuron transfectants.
(A) Total axon length (*left*) and total dendrite length (*right*). *Error bars*, SD. *, $p < 0.05$; **, $p < 0.01$; ***, $p < 0.001$ in ANOVA and post-hoc Dunnett test, compared to WT values.
(B) The results of Sholl analysis for dendrites. *Error bars*, SEM. *, $p < 0.05$; ***, $p < 0.001$ in two-way repeated measures ANOVA and post-hoc Dunnett test, compared to WT values (10–120 μm radius range). †, $p < 0.05$; †††, $p < 0.001$ in two-way repeated measures ANOVA and post-hoc Dunnett test, compared to empty vector values (10–120 μm radius range).
n = 26–36 cells for each expression construct.

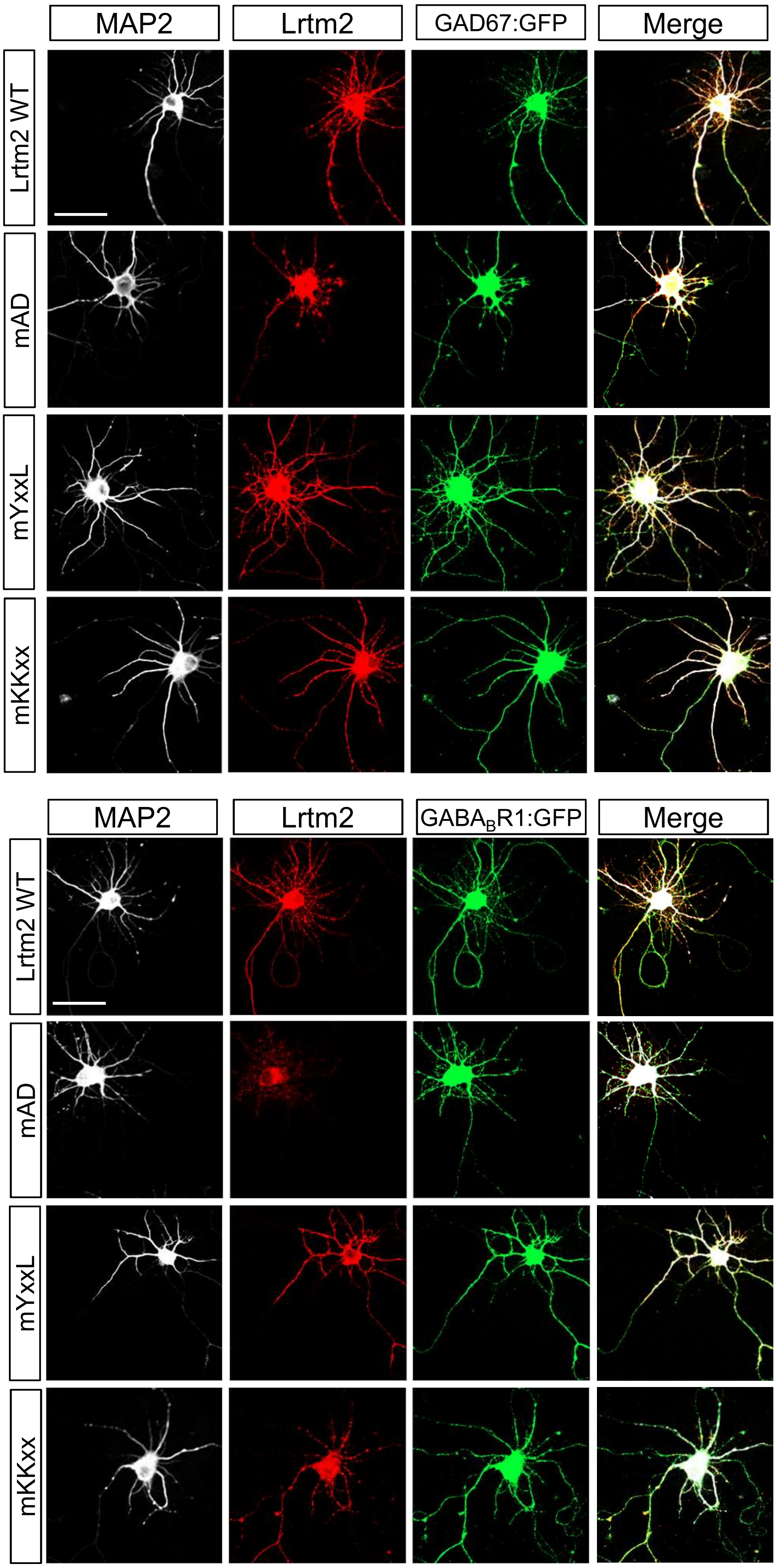

Supplementary Figure 13. Representative images for MAP2, GAD67:GFP double immunostaining (*top*), and for MAP2, $GABA_BR1$:GFP double immunostaining (*bottom*). MAP2-positive areas are shown in *white* in the left column. Lrtm2 WT and mutants are detected by anti-Lrtm2 antibody (*red*). GAD67:GFP or $GABA_BR1$:GFP is detected by anti-GFP antibody (*green*). Scale bars, 50 µm.

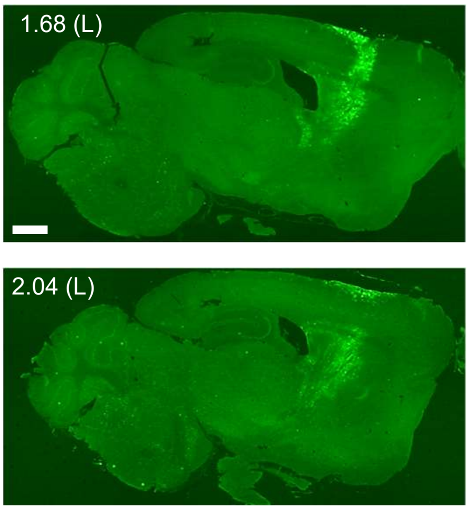

Supplementary Figure 14. GAD67:GFP distribution 4 days after the virus vector injection. GAD67:GFP expressing AAV is injected into the right dorsal striatum of adult Lrtm2 WT mouse. GFP is detected by anti-GFP antibody (*green*) 4 days after the injection. The immunopositive signals can be detected widely in the dorsal striatum. Scale bars, 50 µm.
